# The Video Manipulation Effect (VME): A quantification of the possible impact that the ordering of YouTube videos might have on opinions and voting preferences

**DOI:** 10.1371/journal.pone.0303036

**Published:** 2024-11-20

**Authors:** Robert Epstein, Alex Flores

**Affiliations:** American Institute for Behavioral Research and Technology, Vista, California, United States of America; Satyawati College (Eve.), University of Delhi, INDIA

## Abstract

Recent research has identified a number of powerful new forms of influence that the internet and related technologies have made possible. Randomized, controlled experiments have shown, for example, that when results generated by search engines are presented to undecided voters, if those search results favor one political candidate over another, the opinions and voting preferences of those voters can shift dramatically–by up to 80% in some demographic groups. The present study employed a YouTube simulator to identify and quantify another powerful form of influence that the internet has made possible, which we have labeled the Video Manipulation Effect (VME). In two randomized, controlled, counterbalanced, double-blind experiments with a total of 1,463 politically-diverse, eligible US voters, we show that when a sequence of videos displayed by the simulator is biased to favor one political candidate, and especially when the “up-next” video suggested by the simulator favors that candidate, both the opinions and voting preferences of undecided voters shift dramatically toward that candidate. Voting preferences shifted by between 51.5% and 65.6% overall, and by more than 75% in some demographic groups. We also tested a method for masking the bias in video sequences so that awareness of bias was greatly reduced. In 2018, a YouTube official revealed that 70% of the time people spend watching videos on the site, they are watching content that has been suggested by the company’s recommender algorithms. If the findings in the present study largely apply to YouTube, this popular video platform might have unprecedented power to impact thinking and behavior worldwide.

## 1. Introduction

More than 5 billion people now have internet access, with these individuals potentially susceptible to efforts from tech companies to influence their thinking and behavior. Our research team has discovered and quantified several of these techniques in randomized, controlled experiments conducted since 2013. Our research demonstrates that some new types of manipulation that the internet has made possible can easily shift votes and opinions without people’s knowledge and without leaving paper trails for authorities to trace [1–7]. A growing body of non-scientific evidence suggests that Big Tech companies might sometimes use these new techniques of influence strategically and deliberately [8–12, cf. 13]. Below, we will give some examples of such evidence. In every instance, we acknowledge (a) that the industry professionals we quote are stating their opinions, (b) that we are relying in many instances on credible news sources, not academic journals, and (c) that the assertions made are not based on peer-reviewed research. We will leave it to the reader to decide how seriously to take this information. We offer it simply to put our present research into a larger social context.

### 1.2 Possible concerns about Big Tech influence

Tristan Harris, a former “design ethicist” at Google, has stated publically that he was a member of a team at the company whose job it was to influence “a billion people’s attention and thoughts every day” [14]. Jaron Lanier, one of the early investors in Google and Facebook, claims that Big Tech content has “morphed into continuous behavior modification on a mass basis” [14]. Another early investor in these companies, Roger McNamee, has said that he now regrets having supported them, asserting that they now constitute “a menace to public health and to democracy” [15, cf. 14].

Three recent leaks of internal content from Google might also be cause for concern. First, in emails leaked from the company to *The Wall Street Journal* in 2018, employees were discussing how they might be able to change people’s views about Trump’s travel ban by using what they called “ephemeral experiences” [8]–that is, content such as search results and newsfeeds which appears briefly, impacts the user, and then disappears forever. Second, a 9-min video called “The Selfish Ledger” described the power that the company has to “sequence” human behavior “towards a desired result” according to “Google’s values” [16, cf.^17^]. Third, “The Good Censor,” a company PowerPoint presentation, explained that tech companies had been forced over the years to move away “from passive facilitation to active curation” of content, deciding what content users worldwide could and could not see [18].

In recent years, a number of authorities and experts have expressed particular concern about the way Google’s YouTube platform might be influencing users, especially young children [19–26, cf. 27–36]. A 2019 *New York Times* investigation concluded that “YouTube’s algorithms may have played a decisive role” in the rise of right-wing Brazilian president Jair Bolsonaro by “boost[ing] fringe videos into the mainstream” and helping to spread conspiracy theories and misinformation, especially about diseases [37]. In some cases, when users in Brazil were watching sports videos, YouTube’s up-next suggestion (normally, the video image shown in the upper-right of the screen) would be for a Bolsonaro video, with one Bolsonaro video leading to others [29]. In some instances, just a few clicks have been known to take users down “rabbit holes” of similar videos making extreme claims that have sometimes radicalized them [20, 28, 29, 35–40, cf. 41–43]. This phenomenon has prompted sociologist Zeynep Tufekci to label YouTube as “one of the most powerful radicalizing instruments of the 21st century” [29, 34].

One of the most interesting cases of radicalization, reported in 2019, involved a 26-year-old White male named Caleb Cain of West Virginia–“a college dropout looking for direction” [28]. He turned to YouTube for guidance and was soon “pulled into a far-right filled universe, watching thousands of videos filled with conspiracy theories, misogyny and racism.” He watched more than 12,000 such videos, falling, he later said, into “the alt-right rabbit hole.” Cain’s conversion apparently did not cause him to act violently, but other converts have been more aggressive. In 2020, Brenton Harrison Tarrant, a 28-year-old White male from Australia, was convicted of 51 counts of murder, 40 counts of attempted murder, and one count of terrorism–the first terrorist convicted in New Zealand’s history. His rampage took place in March, 2019, and his victims were worshippers at two mosques in Christchurch. Tarrant had been radicalized by YouTube videos. He even had made use of infinitelooper.com, which would repeat certain inspirational YouTube videos for him endlessly [44].

In 2022, the Anti-Defamation League published an ambitious study–well executed but not peer-reviewed, as far as we can tell–on user exposure to “alternative” and extremist content on YouTube [45]. Based on data obtained from a representative sample of 859 people in the US who were enrolled with YouGov, a national polling firm, the study concluded that roughly 1 in 5 YouTube users are exposed to “alternative” content–“channels that can serve as gateways to more extreme forms of content”–and that 1 in 10 users are exposed to extremist content directly. Although the researchers did not find evidence that extreme or disturbing YouTube content converted people with moderate views, they did find (a) that such content strongly attracted people “who already have high levels of racial resentment,” (b) that when people watch such videos, they are “more likely to see and follow recommendations to similar videos,” and (c) that when someone is viewing an extremist video, other extremist videos are likely to be recommended alongside them [45].

Some studies have found stronger evidence of radicalization on YouTube [46, 47, cf. 43, 48–53]. A large-scale 2020 study published by the Association for Computing Machinery that examined more than 330,000 videos concluded, for example, “We find strong evidence for radicalization among YouTube users, and that YouTube’s recommender system enables Alt-right channels to be discovered, even in a scenario without personalization…. Moreover, regardless of the degree of influence of the recommender system in the process of radicalizing users, there is significant evidence that users are reaching content sponsoring fringe ideologies from the Alt-lite [people who ‘flirt with’ white supremacist ideology] and the Intellectual Dark Web” [46].

Other recent studies have catalogued and counted the growing number of extremist videos available on the YouTube platform [42, 46–50, 54–57]. Even though YouTube regularly removes many such videos from its platform, the number of alternative and extremist videos available to users worldwide on any given day is probably in the millions. This conjecture is based on recent surveys suggesting that upwards of 20% of internet users in the US have encountered hateful or harassing content on YouTube, which, at this writing (July 4, 2023), hosts more than 800 million videos [45, 58, 59]. The disturbing content could be in the videos themselves or in the comments provoked by those videos.

### 1.3 The value of controversial and addictive content

Both leaks and official statements from Big Tech platforms suggest that controversial content is an important part of content offerings because (a) it draws more traffic, and more traffic is generally more profitable [14, 34, 60, 61, cf. 35, 49], (b) it keeps people on a website longer [14, 35, 63, cf. 29, 37], and (c) it increases the “watch time” of videos [14, 35, cf. 29, 37]. Content personalization on platforms like YouTube has proved to be especially important in increasing the “stickiness” of websites [14, 28, 39, 62–64]. Even Mark Zuckerberg, the CEO of Facebook/Meta, acknowledged the value of controversial content from a business perspective in an official statement he released in 2018. According to Zuckerberg, “Our research suggests that no matter where we draw the lines for what is allowed, as a piece of content gets close to that line, people will engage with it more on average–even when they tell us afterwards they don’t like the content” [65].

Multiple studies have also shown that YouTube’s recommender algorithms are especially aggressive in recommending “pseudoscientific” videos and other content of dubious value [21, 22, 62, 66, cf. 67]; again, the more dubious the content, the more traffic is generated and the more watch time is increased. Anti-vaccine videos on YouTube have been shown to lead to recommendations of a disproportionately large number of additional anti-vaccine videos compared to pro-vaccine videos [68].

Even if radicalization on YouTube were rare, YouTube’s video-management algorithm does allow it to occur. A vulnerable individual can be drawn into a highly persuasive sequence of videos when three important mechanisms are in alignment: filtering, ordering, and customization. Filtering is the process by which the algorithm selects some videos for presentation (a small sample) and rejects others (the vast majority). Ordering is the process by which the algorithm places one video ahead of another. And customization is the process by which the algorithm refines the filtering and ordering based on (a) information from the personal profile that Google has accumulated about the user and (b) priorities that the company or its employees might have about how they want to influence users. When these factors align, users can be caught in so-called “loops,” “echo chambers,” and “filter bubbles” of similarly biased content [48, 50, 69–78, cf. 79–83]. Relevant here is the fact that YouTube’s algorithms also determine whether content goes viral on the platform [84, 85].

Note that all three of these factors also operate on Google’s search engine. Although we tend not to think about YouTube this way, YouTube is actually the second largest search engine in the world, as well as the world’s largest video-sharing social media platform [86]. The first video a user watches during a YouTube session is usually suggested after the user types a search term into YouTube’s search bar. After that first search is completed, however, YouTube and the Google search engine part ways in how they influence the user. On Google, the user at some point clicks away to another website–ideally, from a business perspective, to a website Google wants the user to visit [4]. As Larry Page, co-founder of Google said famously long ago, “We want to get you out of Google and to the right place as fast as possible” [87]. Whether he meant the right place for the user or the right place for the company is unclear. On YouTube, the goal is the opposite: It is to keep the user on the platform as long as possible [14, 28, 29, 34, 35]. That behavioral addiction is the goal has been acknowledged by Google whistleblowers [14, 88, 89] and suggested by researchers [90, 91].

YouTube generally accomplishes these ends in ways that are too subtle for most users to discern. Users are likely aware that if they fail to search for another video or to click on one of the recommended videos (shown to the right of or just below the video screen), YouTube will automatically play another video–the up-next video shown on desktop and laptop computers in the upper-right corner of the computer screen. On smart phones, the up-next video will often play automatically even if the user has never seen the thumbnail version of it; this can occur, for example, when the phone is tilted to the landscape (horizontal) position, which causes the video that is playing to take up the entire screen. Until January 2015, a labeled “autoplay” on-off switch appeared above the up-next video on desktop and laptop computers which allowed the user to stop up-next videos from playing automatically. At this writing, however, the switch appears immediately below the video, and it no longer has a label on it; one must scroll over it (on desktop and laptop computers) or touch it (on mobile devices) even to find out what the button is for. These cosmetic changes were likely implemented to increase watch time [92–94, cf. 95–97]. To view images of YouTube’s autoplay on-off switch pre- and post-January 2015, visit https://aibrt.org/downloads/VME_PLOS_2024/S1_Fig.jpg and https://aibrt.org/downloads/VME_PLOS_2024/S2_Fig.jpg.

According to official YouTube statements, watch time is YouTube’s most important concern [61, 98, cf. 14]. In 2018, a YouTube official revealed that 70% of the time people spend watching videos on the site, they are watching content that has been suggested by the company’s up-next algorithm [99]. The importance of watch time has also been emphasized in public statements by former Google software engineer Guillaume Chaslot, who summarized this issue thus: “Watch time was the priority. Everything else was considered a distraction” [96]. When Chaslot suggested to his supervisors that the YouTube algorithm be modified to free users from content feedback loops, they rejected his ideas. “[T]he entire business model is based on watch time,” according to Chaslot, and “divisive content” is especially effective in locking in user attention [14, cf. 39]. Tristan Harris expressed this concept metaphorically: “There’s a spectrum on YouTube between the calm section–the Walter Cronkite, Carl Sagan part–and Crazytown, where the extreme stuff is. If I’m YouTube and I want you to watch more, I’m always going to steer you toward Crazytown” [28]. Unfortunately, such content can include “bizarre and disturbing” content directed at young children [100].

### 1.4 YouTube’s power to influence users

According to various industry and news reports, YouTube exercises its power to influence and control by (a) demonetizing content it finds objectionable and thus discouraging certain content creators from posting videos [102–104, cf. 35, 101, 105], (b) restricting access to videos, in one case limiting access to more than 50 videos from the conservative Prager University organization, among them a video by noted Harvard Law School professor Alan Dershowitz about the founding of Israel and a video about the “thou shall not kill” provision of The Ten Commandments [106, 107, cf. 101], (c) deleting videos from its platform [108–112], and (d) reordering videos–in other words, boosting the positions of videos it is trying to promote and demoting videos it is trying to suppress [37, 57, 63, 64, 66]. In a 2-minute 2017 video leaked from Google in 2019 by a former Google staffer, Susan Wojcicki, then CEO of YouTube, explains to her staff the process by which YouTube’s recommender algorithm was currently being altered to boost content the company viewed as valid and demote content the company considered suspect [113]. To view the full video, visit https://vimeo.com/354354050. That re-ranking process has continued to this day; at this writing (June 28, 2023), US Congressman and Presidential candidate Robert F. Kennedy Jr. is in the news protesting the removal of several of his videos from YouTube [114, 115].

When users have challenged such actions, US courts have repeatedly ruled in favor of Google and YouTube, asserting that by deleting or reordering content, these platforms, as private companies, are exercising their right to free speech under the First Amendment to the US Constitution [106, 116–119].

A growing body of research demonstrates the power of YouTube’s recommender algorithms, either to cause people to formulate opinions where their opinions are initially weak, or to further strengthen opinions where opinions are initially strong [66, 71–74, cf. 78, 120–122]. Some of this research extends these general findings to the political realm [123]. For example, a 2020 study by Cho et al. (conducted with 108 undergraduate students at one university) demonstrated the power that YouTube’s recommender algorithms have to “reinforce and polarize” existing political opinions [76, cf. 77]. Newer studies suggest that YouTube’s up-next algorithm might be biased to some extent in one direction politically [43, 81], although “communities” of YouTube users can have almost any political bias [50, 120].

Whether videos are generally more influential than auditory, textual, or still-image media is a matter that has not been well explored, to our knowledge–in part, we believe, because of the difficulties inherent in designing studies that compare the persuasiveness of these media fairly. One recent study suggests, however, that video is substantially more powerful than text in convincing people that political content is real, but that it is only slightly more persuasive than text [124, 125, cf. 126–131]. Whatever the truth is about direct comparisons, researchers have consistently found that videos get far more “shares” online than other forms of media do–according to one recent estimate, “1200% more shares than text and images combined” [132, cf. 133], and online content apparently has far more impact, in general, than offline content [134, cf. 123].

### 1.5 The importance of capturing ephemeral content

These new methods of influence are especially problematic because they are controlled worldwide (outside the People’s Republic of China) by a small number of corporate monopolies, which means one cannot counteract them. If a political candidate airs an attack ad on television or on the internet, the opponent can air a rejoinder. But if a large online platform uses new techniques of influence to support a candidate, the opponent can do nothing to counteract that influence; in many cases, that manipulation might not even be visible. As we noted earlier, many online manipulations also make strategic use of ephemeral experiences to change thinking or behavior; that normally guarantees that these manipulations leave no paper trails for authorities to trace. Note that although YouTube videos are *not* ephemeral, the video sequences and up-next suggestions the company makes are indeed ephemeral. They are generated on the fly for the individual user and stored nowhere, and there is no way, to our knowledge, for anyone–including Google employees–to go back in time to regenerate them.

We and our colleagues have successfully built monitoring systems that have preserved increasingly larger bodies of ephemeral experiences in the days leading up to six elections in the US [135–137]. In 2020, we preserved and analyzed more than 1.5 million ephemeral experiences obtained and then aggregated through the computers of a politically-diverse group of 1,735 registered voters in four swing states. We captured data on the Google, Bing, and Yahoo search engines, as well as on YouTube and Google’s home page [138, 139], and we found substantial political bias on these platforms, sufficient, perhaps, to have shifted millions of votes among undecided voters. Based on our preliminary analysis of data we had collected, on November 5, 2020, three US Senators sent a warning letter to the CEO of Google about the political bias we had detected in Google content, and Google immediately turned off political bias in the search results it was sending to Georgia residents in the weeks leading up to the two US Senate runoff elections scheduled there for January 5, 2021. Google also stopped sending go-vote reminders to Georgia residents. Monitoring systems, it appears, can be used to make Big Tech companies accountable to the public. As Supreme Court Justice Louis D. Brandeis opined a century ago, “Sunlight is said to be the best of disinfectants; electric light the most efficient policeman” [140].

In 2022, we expanded our network of “field agents” to include 2,742 registered voters, and we preserved more than 2.5 million ephemeral experiences on multiple platforms, this time including both Twitter and Facebook [141]. We are currently in the process of building a permanent, large-scale “digital shield” in all 50 US states which will, we hope, protect our elections from manipulation by emerging technologies for the foreseeable future [141–143]. At this writing (March 20, 2024), this automated system is preserving and analyzing ephemeral online content 24 hours a day from the computers of a politically-balanced group of more than 14,000 registered voters in all 50 US states [144], and we have thus far preserved more than 80 million ephemeral experiences.

Proposals have also been made to try to track or reduce the potential manipulative power of software such as YouTube’s recommender algorithms by developing methods that increase algorithmic transparency and accountability [145–149, cf. 150]. The companies that control these algorithms will likely resist such efforts, however, and because of their increasing reliance on machine learning techniques, algorithms have grown increasingly opaque over the years–so mysterious that even the original programmers can’t understand them [151]. The clearest way, in our view, to monitor, preserve, and analyze algorithmic output is to look over the shoulders of large, representative samples of real users as they are viewing real content. Doing so is necessary in part because so much content is now customized to fit characteristics of individual users [152–154].

### 1.6 Video Manipulation Effect (VME)

The present paper focuses on a powerful new form of influence we call the Video Manipulation Effect (VME), in which we use a YouTube simulator we call DoodleTube to determine the extent to which we can shift the opinions and voting preferences of undecided voters by manipulating the order of recommended videos–in other words, by exercising control over YouTube’s recommender algorithms. The videos were biased to favor one candidate or his opponent. By manipulating the order, we also had control over which video was in the up-next position and which therefore would play automatically if the user did not select a different video. By parsing the data demographically, we also determined how vulnerable people in different demographic groups were to the manipulation.

## 2. Experiment 1: Biased video ordering with no mask

In our first experiment, we sought to determine whether a biased ordering of recommended videos–biased to favor one political candidate–could shift opinions and voting preferences toward that candidate. By “no mask,” we mean that high-ranking videos consistently favored one candidate. In Experiment 2, in order to reduce perception of bias, we masked the bias by mixing in videos that supported the non-favored candidate (see Procedure sections below for details).

### 2.1 Methods

#### 2.1.1 Ethics statement

The federally registered Institutional Review Board (IRB) of the sponsoring institution (American Institute for Behavioral Research and Technology) approved this study with exempt status under US Health and Human Services (HHS) rules because (a) the anonymity of participants was preserved and (b) the risk to participants was minimal. AIBRT is registered with the HHS Office for Human Research Protections (OHRP) under IORG0007755. The IRB is registered with OHRP under number IRB00009303, and the Federalwide Assurance number for the IRB is FWA00021545. Informed written consent was obtained for both experiments as specified in the Procedure section of Experiment 1.

#### 2.1.2 Participants

After cleaning, our participant sample for this experiment consisted of 959 eligible US voters recruited through the Amazon Mechanical Turk (MTurk) subject pool. During the cleaning process, we removed participants who reported an English fluency level below 6 on a 10-point scale, where 1 was labeled “Not fluent” and 10 was labeled “Highly fluent.” In order to assure that our participants were undecided, we also removed participants who reported a level of familiarity with either of the two candidates exceeding 3 on a 10-point scale. In all, 41 participants were removed during cleaning.

Overall, 558 (58.2%) of our participants identified themselves as female, 391 (40.8%) as male, and 10 (1.0%) chose not to identify their gender. Racial and ethnic background was as follows: 701 (73.1%) of our participants identified themselves as White, 102 (10.6%) as Black, 75 (7.8%) as Asian, 58 (6.0%) as Mixed, and 22 (2.3%) as Other. Overall, 26.8% of the individuals in the sample identified themselves as non-White.

Regarding level of education completed: 2 (0.2%) of our participants reported no education; 41 (4.3%) reported not having a high school degree; 309 (32.2%) reported completing high school; 428 (44.6%) reported having a bachelor’s degree; 148 (15.4%) reported having a master’s degree; and 31 (3.2%) reported having a doctoral degree.

Regarding political alignment: 435 (45.4%) of our participants identified themselves as liberal, 291 (30.3%) as moderate, 187 (19.5%) as conservative, 30 (3.1%) as not political, and 16 (1.7%) as other.

Regarding YouTube usage: 958 (99.9%) of our participants reported that they have used YouTube before, and 602 (62.8%) reported that they have used YouTube to get information about political or ideological topics. Participants reported using YouTube an average of 18.0 (*SD* = 32.4) times a week.

See S1 Table for detailed demographic information for Experiment 1.

#### 2.1.3 Procedure

All procedures were conducted online, with sessions conducted on December 7, 2021, December 11, 2021, and January 7, 2022. Participants were first asked two screening questions; sessions were terminated if they said they were not eligible to vote in the US or if they reported a level of familiarity with Australian politics exceeding 3 on a 10-point scale. We chose to exclude people who were highly familiar with Australian politics in order to assure that our participants–all from the US–were likely to be undecided about which candidate to vote for in the election we featured in the experiment: the 2019 election for Prime Minister of Australia.

Participants who passed our screening questions were then asked various demographic questions and then given instructions about the experimental procedure. We also displayed a short video and asked participants whether they were able to see the video and whether the video autoplayed. If the videos did not play at all or did not autoplay the session was terminated. At the end of the instructions page, and in compliance with American Psychological Association and HHS guidelines, participants were asked for their consent to participate in the study. If they clicked “I Do,” the session continued; if they clicked “I Do Not,” the session ended. Participants were then asked further questions about their political leanings and voting behavior.

Participants were then given a short paragraph about each of two candidates who ran for Prime Minister of Australia in 2019, each about 120 words in length (see S1 Text in Supporting Information for the full paragraphs). Participants were next asked three opinion questions on a 10-point scale about each candidate: one regarding their overall impression of the candidate, one regarding how likeable they found the candidate, and one regarding how much they trusted the candidate. They were then asked, on an 11-point scale with values ranging from 5 to 0 to 5, which candidate they would be likely to vote for if they “had to vote today.” Finally, they were asked which candidate they would vote for if they “had to vote right now” (forced choice).

Participants were then given an opportunity to use DoodleTube–our YouTube simulator–to watch videos about these candidates in order to gather information to help them decide which of the two candidates to vote for. They were given a maximum of 15 minutes and a minimum time of 10 minutes to view the videos. See S2 Text for the complete instructions.

**Fig 1:**
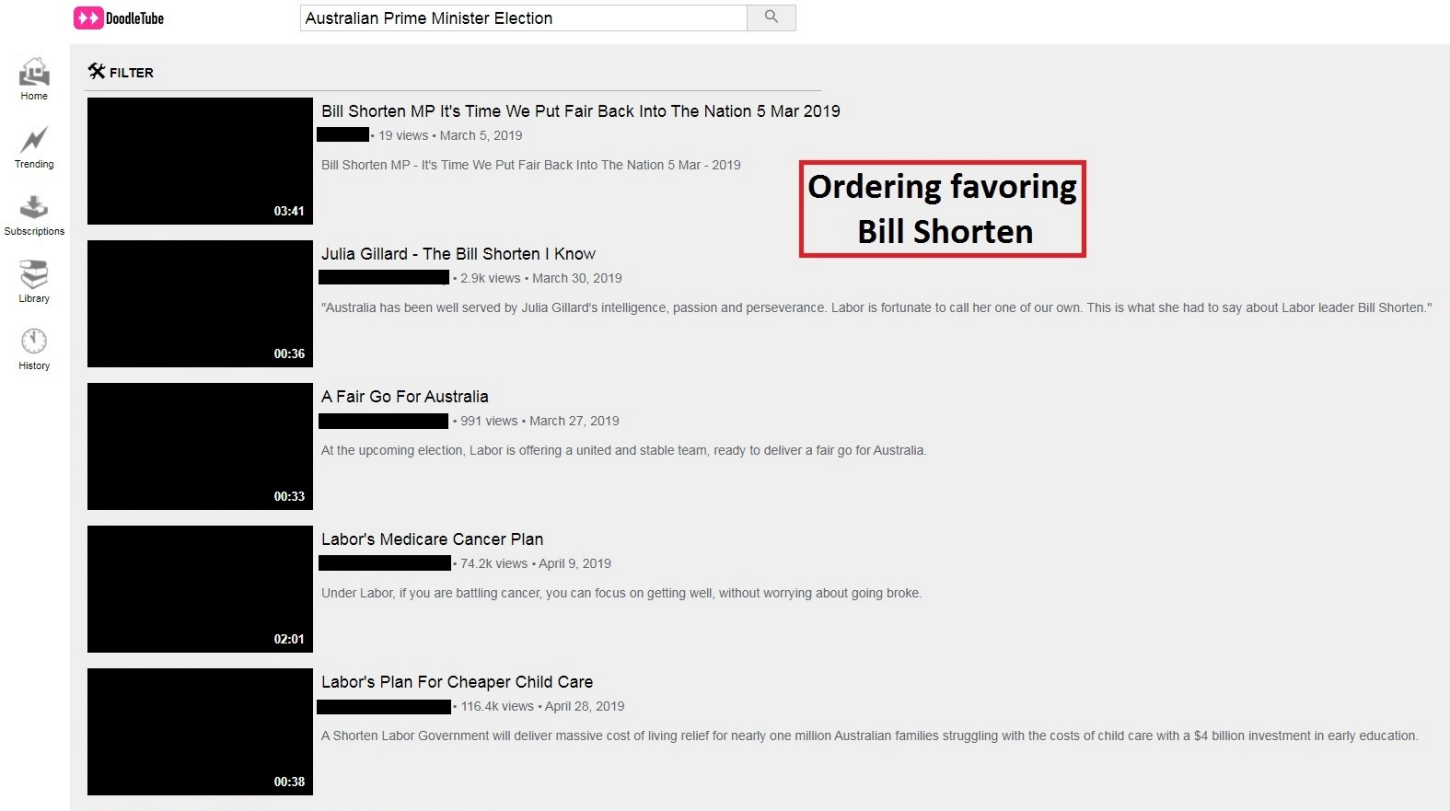
Initial screen when a DoodleTube session begins, with identifying images and information removed. In this instance, the participant had been randomly assigned to a group in which the order of the videos favored candidate Bill Shorten. The red-outline box above was not shown. To view the unblocked image, visit https://aibrt.org/downloads/VME_PLOS_2024/Fig1.jpg.

**Fig 2:**
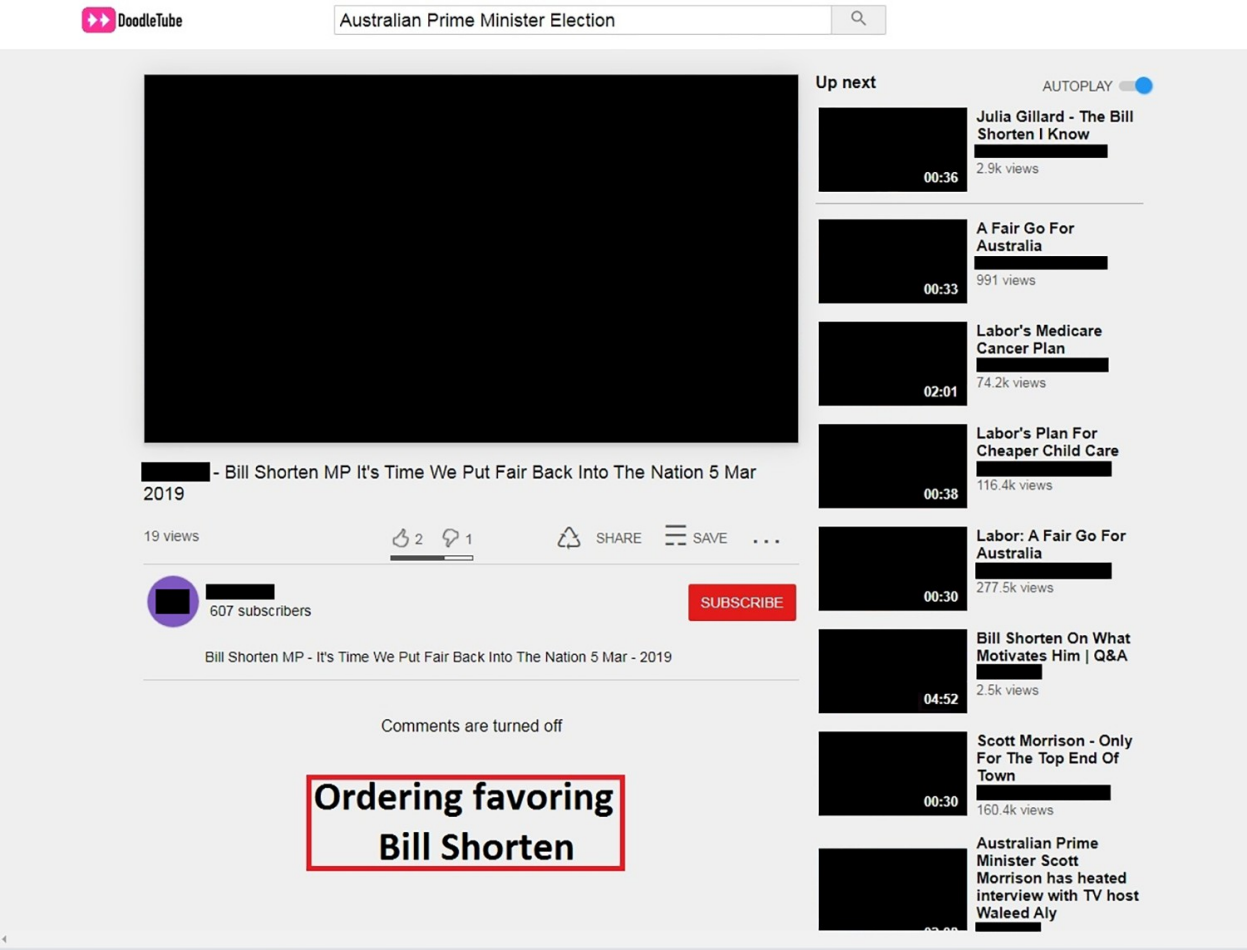
Screen that appears after the participant has clicked one of the videos shown in Fig 1, with identifying images and information removed. The red-outlined box above was not shown. To view the unblocked image, visit https://aibrt.org/downloads/VME_PLOS_2024/Fig2.jpg.

On the next screen, participants saw an online video platform called DoodleTube displaying a search bar with a pre-inputted query of “Australian Prime Minister Election” and a series of videos relating to that query (Fig 1). Participants could click on any of the videos to play it. When a video was clicked, the screen switched to a video view screen with the up-next video on the top of the right side bar, along with other recommended videos beneath it (Fig 2).

The participant could watch an entire video, or the participant could click on a different video to switch the view to that new one. The participant could also allow a video to play to the end and then allow the up-next video to play; this occurred automatically if the participants did not click on another video.

**Fig 3:**
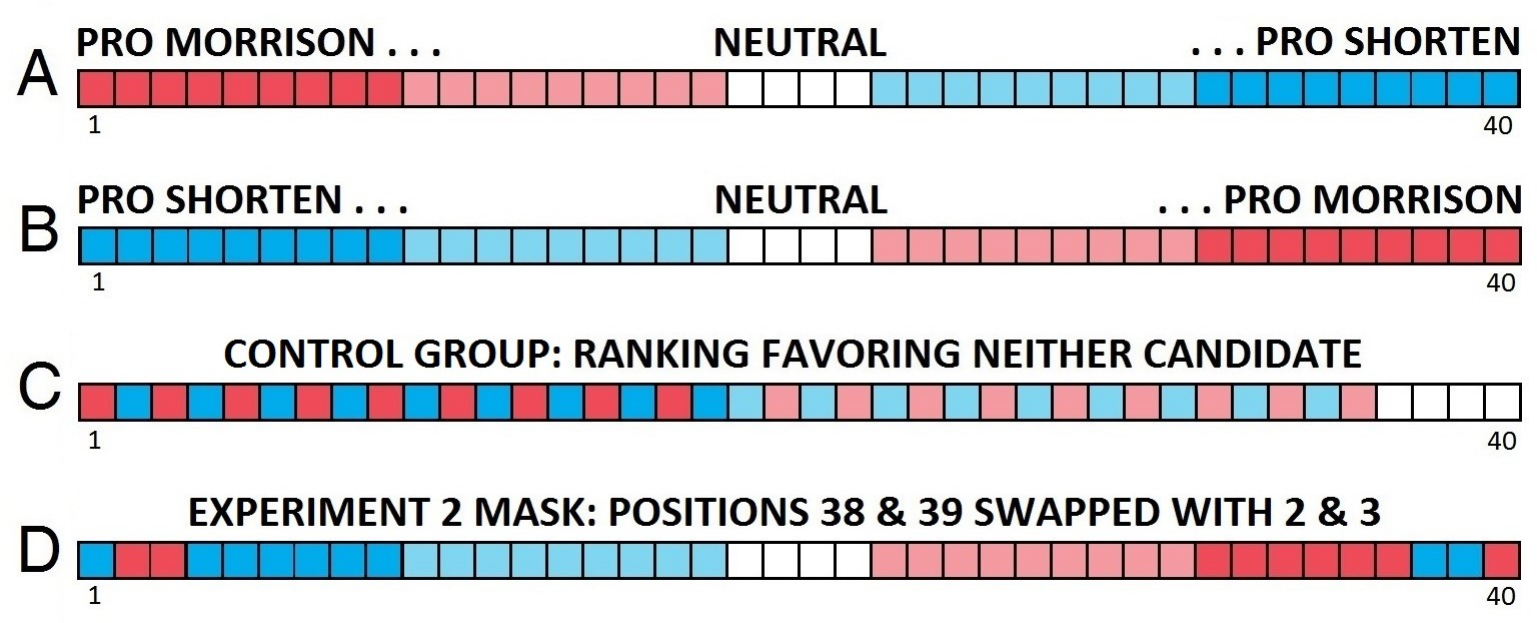
Ordering of videos in Experiments 1 and 2. A: Experiment 1, Group 1 (first bias group, 40 videos in order from Pro Morrison to neutral to Pro Shorten). B: Experiment 1, Group 2 (second bias group, 40 videos in order from Pro Shorten to neutral to Pro Morrison). C: Group 3 (control group, 40 videos in which Pro Morrison and Pro Shorten videos are alternated). D: Experiment 2: The manipulation is masked by swapping the video in Position 2 with the video in Position 39, and the video in Position 3 with the video in Position 38 (shown only for Group 1, Pro Morrison bias group).

Participants had been randomly assigned to one of three groups: Pro-Candidate-A (Scott Morrison), Pro-Candidate-B (Bill Shorten), or the control group. People in all three groups had access to all 40 of the videos that were included in the experiment, but the videos were listed in a different order in each group. As shown in Fig 3A, in the Pro-Morrison group, the order of the videos would go from Pro-Morrison videos to Pro-Shorten videos. If the participant was assigned to the Pro-Shorten group, the order of the videos would go from Pro-Shorten videos to Pro-Morrison videos (Fig 3B). The control group would have both Pro-Morrison and Pro-Shorten videos in alternation (Fig 3C).

The End button in the upper-left corner of the web page was invisible and inaccessible for the minimum required video view time of 10 minutes. After the 10 minutes was up, the End button appeared. However, participants were allowed to continue browsing the videos up to the maximum time of 15 minutes. When the 15 minutes was up, the participants were redirected to the questions page. The timer was paused when the videos were paused and restarted when the videos were restarted to ensure participants were viewing the video for the required amount of time. The autoplay feature was on by default and could not be turned off. Users could, however, go to whatever video they desired either on the sidebar or the home screen.

Following the DoodleTube experience, participants were again asked the same series of questions that they were asked before they began watching the videos: three opinion questions about each candidate (10-point scale on impression, likeability, and trust), which candidate they were likely to vote for (11-point scale from 5 to 0 to 5), and which candidate they would vote for now (forced choice).

Next, participants were asked whether any of the content they had seen on DoodleTube “bothered” them in any way. They could reply Yes or No, and then they could explain their answer by typing freely in a text box. This is a conservative way of determining whether people perceived any bias in the content they had seen. We could not ask people directly about their awareness of bias because leading questions of that sort often produce misleading answers [155]. To assess bias we searched the textual responses for words such as “bias,” “skewed,” or “slanted” to identify people in the bias groups who had apparently noticed the favoritism in the search results they had been shown.

### 2.2 Results

For election campaign officials, the most important result in this experiment would almost certainly be what we call the “vote manipulation power” or VMP, which we define as the post-manipulation percentage increase in the number of participants preferring the favored candidate (in response to the forced-choice question). In a group that is initially split 50/50, the VMP also turns out to be the post-manipulation vote margin. For additional information about VMP and how we compute it, see S3 Text in the Supporting Information.

In Experiment 1, for all participants in the two bias groups combined (Groups 1 and 2), the VMP was 51.5% (McNemar’s Test *X*^*2*^ = 98.20, *p* < 0.001). S2–S4 Tables show VMPs broken down by gender, race/ethnicity, and level of educational attainment. For those demographic characteristics, we found significant and consistent effects only for gender, with females having higher VMPs than males.

On the 11-point voting preferences scale, pre-manipulation, we found no significant difference between mean ratings in the three groups (*M*_Morrison_ = -0.22, *SD* = 2.75; *M*_Shorten_ = -0.15, *SD* = 2.90; *M*_Control_ = 0.04, *SD* = 2.81; Kruskal-Wallis *H* = 1.53; *p* = 0.47 NS). Post manipulation, we found a significant difference between mean ratings in the three groups (*M*_Morrison_ = -1.85, *SD* = 3.19; *M*_Shorten_ = 2.14, *SD* = 3.11; *M*_Control_ = 0.45, *SD* = 3.53; *H* = 188.67; *p* < 0.001). Participants in Group 1 shifted 1.63 points towards the favored candidate (Morrison), and participants in the Group 2 condition shifted 2.29 points towards the favored candidate (Shorten). In addition, the pre-manipulation mean preference for the favored candidate (Groups 1 and 2 combined) was significantly different from the post-manipulation mean preference for the favored candidate (Groups 1 and 2 combined) (*M*_Pre_ = 0.04, *SD*_Pre_ = 2.83, *M*_Post_ = 1.99, *SD*_Post_ = 3.15, Wilcoxon *z* = -12.02, *p* < 0.001). Opinion ratings for both candidates also shifted significantly in the predicted direction (Table 1). Finally, the proportion of videos our participants watched which were selected by our up-next recommendation was 56.8% (*SD* = 27.1). (Nonparametric statistical tests such as the Kruskal-Wallis H are frequently employed in this study because the ratings of the candidates lie on ordinal scales [156]. Means and standard deviations in such instances are reported for comparison purposes, although the appropriateness of their use with ordinal data has long been debated [157, 158]).

**Table 1:** Experiment 1: Pre and post opinion ratings of favored and non-favored candidates.

	Favored Candidate Mean (SD)			Non-Favored Candidate Mean (SD)			
	Pre	Post	Diff	Pre	Post	Diff	z^†^
Impression	7.07 (1.87)	7.42 (2.35)	0.35	7.01 (1.93)	4.82 (2.45)	-2.19	-13.64***
Trust	6.18 (2.07)	6.70 (2.52)	0.52	6.22 (2.07)	4.52 (2.39)	-1.70	-12.84***
Likeability	6.98 (1.85)	7.43 (2.41)	0.45	6.95 (1.96)	4.81 (2.50)	-2.14	-14.12***

^†^z-score represents Wilcoxon signed ranks test comparing post-minus-pre ratings for the favored candidate to the post-minus-pre ratings for the non-favored candidate

*** *p* < 0.001

Demographic breakdowns of the data obtained on the 11-point voting preference scale in Experiment 1 are shown in S5–S13 Tables. Male/female differences on this scale were highly significant (S5–S7 Tables). Differences by educational attainment and race/ethnicity were not consistently significant (S8–S13 Tables).

Although the shift in voting preferences was substantial in Experiment 1 (VMP = 51.5%), it is notable that 33.0% of the participants in the two bias groups (Groups 1 and 2) appeared to detect political bias in the videos they watched. In SEME experiments, perception of bias can easily be reduced with masking procedures–for example, by mixing one or more search results favoring the non-favored candidate mixed among the more frequent and higher-ranking search results favoring the other candidate [2]. Could we reduce perception of bias in a YouTube-like environment using a similar mask, and, if so, might we still be able to produce a substantial shift in voting preferences? We attempted to answer these questions in Experiment 2.

## 3. Experiment 2: Biased video ordering with mask

### 3.1 Methods

#### 3.1.1 Participants

After cleaning, our participant sample consisted of 491 eligible US voters recruited through the MTurk subject pool. The cleaning procedure was identical to that of Experiment 1, and a total of nine participants were removed from the sample during that procedure. The group was demographically diverse. See S1 Table for detailed demographic information for Experiment 2.

Regarding YouTube usage: 489 (99.6%) reported that they had used YouTube before, and 310 (63.1%) reported that they had used YouTube to get information about political or ideological topics. Participants reported using YouTube an average of 16.2 (*SD* = 30.2) times per week.

#### 3.1.2 Procedure

The procedure in Experiment 2, with sessions conducted on December 24, 2021, and January 8, 2022, was identical to that of Experiment 1, with one exception: The order of recommended videos in the experimental groups had a mask in the 2nd and 3rd positions. Specifically, we swapped the usual video in Position 2 with the video from Position 39, and the usual video in Position 3 with the video from Position 38 (see Fig 3D). In other words, in the Pro-Morrison group, the video order remained the same as it was in Experiment 1 except in the 2nd and 3rd positions the videos were Pro-Shorten. Similarly for Pro-Shorten group, the videos in the 2nd and 3rd positions were Pro-Morrison.

### 3.2 Results

In Experiment 1 (no mask), the VMP was 51.5%, and 33.0% of participants in the two bias groups showed some awareness of bias in the ordering of the videos. In Experiment 2 (mask), the VMP was 65.6% (McNemar’s Test *X*^2^ = 67.11, *p* < 0.001), and only 14.6% of participants in the two bias groups showed some awareness of bias in the ordering of the videos. The VMP in Experiment 2 was 27.4% higher than the VMP in Experiment 1 (*z* = 4.23, *p* < 0.001). The perception of bias in Experiment 2 was 55.8% lower than the perception of bias in Experiment 1 (*z* = 6.19, *p* < 0.001). Thus it appears that the manipulation can indeed be masked in such a way as to reduce perception of bias (perhaps to zero) while still producing a substantial shift in voting preferences. Again, S2–S4 Tables show VMPs broken down by gender, race/ethnicity, and level of educational attainment. For those demographic characteristics, we again found consistently significant effects only for gender, with females having higher VMPs than males.

Voting preferences as measured on the 11-point scale also shifted in the predicted direction. Pre-manipulation, we found no significant difference between mean ratings in the three groups (*M*_Morrison_ = -0.08, *SD* = 2.84; *M*_Shorten_ = -0.15, *SD* = 2.65; *M*_Control_ = -0.25, *SD* = 2.88; *H* = 0.27, *p* = 0.873 NS). Post manipulation, we found a significant difference between mean ratings in the three groups (*M*_Morrison_ = -1.87, *SD* = 3.23; *M*_Shorten_ = 2.03, *SD* = 2.88; *M*_Control_ = 0.59, *SD* = 3.53; *H* = 95.64; *p* < 0.001). Participants in Group 1 shifted 1.79 points towards the favored candidate (Morrison), and participants in the Group 2 condition shifted 2.18 points towards the favored candidate (Shorten). In addition, the pre-manipulation mean preference for the favored candidate (Groups 1 and 2 combined) was significantly different from the post-manipulation mean preference for the favored candidate (Groups 1 and 2 combined) (*M*_Pre_ = -0.02, *SD*_Pre_ = 2.75; *M*_Post_ = 1.95, *SD*_Post_ = 3.07; Wilcoxon *z* = -9.05, *p* < 0.001). Opinion ratings for both candidates also shifted significantly in the predicted direction (Table 2). Finally, the proportion of videos our participants watched which were selected by our up-next recommendation was 60.7% (*SD* = 25.6).

**Table 2:** Experiment 2: Pre and post opinion ratings of favored and non-favored candidates.

	Favored Candidate Mean (SD)			Non-Favored Candidate Mean (SD)			
	Pre	Post	Diff	Pre	Post	Diff	*z* ^†^
Impression	6.91 (1.78)	7.30 (2.22)	0.39	6.96 (1.80)	4.89 (2.37)	-2.07	-9.81***
Trust	6.08 (2.01)	6.69 (2.30)	0.61	6.12 (2.03)	4.65 (2.38)	-1.47	-9.06***
Likeability	6.86 (1.81)	7.49 (2.18)	0.63	6.87 (1.79)	4.99 (2.44)	-1.88	-9.91***

^†^z-score represents Wilcoxon signed ranks test comparing post-minus-pre ratings for the favored candidate to the post-minus-pre ratings for the non-favored candidate

*** *p* < 0.001

Demographic breakdowns of the data obtained on the 11-point voting preference scale in Experiment 2 are shown in S5–S13 Tables. Male/female differences on this scale were not consistently significant (S8–S13 Tables). Neither were differences by educational attainment or race/ethnicity (S8–S13 Tables).

## 4. Discussion

Our experiments demonstrate that (a) strategic ordering of videos on a YouTube-like platform can dramatically shift both the opinions and voting preferences of undecided voters, rapidly shifting a substantial portion of them to favor one political candidate (Experiment 1), and (b) this manipulation can be masked to reduce perception of bias while still producing large, predictable shifts in opinions and voting preferences (Experiment 2). These findings are important because, as we have documented in our Introduction, an increasing body of evidence suggests that YouTube itself can have a dramatic impact on the thinking and behavior of people worldwide, sometimes in destructive or self-destructive ways.

Like search results, search suggestions, answer boxes, and newsfeeds, video sequences constructed by YouTube’s recommender algorithm are ephemeral in nature. Once again, that means that this form of influence normally leaves no paper trail for authorities to trace or, perforce, for researchers to study. Without monitoring systems in place to preserve large representative samples of ephemeral content, people will be blind to the ways in which they are being impacted by the algorithms of tech companies, and we will in effect be turning our democracy, and, to some extent, our own minds over to these companies. Some children are especially impressionable [32, 33, 159], and with mobile devices having now become both the babysitter and the companion of choice for children [19, 23], it is reasonable to conjecture that the new forms of influence that the internet has made possible are impacting our children and grandchildren profoundly [20–22, 24–27, 31]. In our view, laws and regulations will never be able to keep pace with rapidly changing technologies, but monitoring systems can. It’s good tech battling bad tech, now and in the future.

In recent months, our team has preserved more than 70 million online ephemeral experiences in all 50 US states through the computers of a politically-balanced group of more than 13,000 registered voters, and we are now beginning to preserve content from the mobile devices of more than 2,000 children and teens (with the permission of their parents). We are also developing ways of analyzing much of this data in real time, and we recently began giving both the authorities and the general public free access to our findings 24 hours a day through a public dashboard [145]. In our view, in a world in which unprecedented power has been given to private companies to impact people’s thinking and behavior, monitoring systems are not optional. Without them, we will not only have no idea how such companies might be influencing us, governments that implement laws or regulations to contain the power of these companies will have no reliable way of measuring compliance with those laws and regulations.

### 4.1 Limitations and future research

Our current procedures do not include any follow up, so we have no way of measuring how long the changes our procedures produce in opinions and voting preferences will last. Just as the content our participants see is fleeting, so might be the changes we are detecting in their opinions and voting preferences. That said, we might actually be underestimating the possible power of VME as it might be impacting real users, because we are exposing our participants to only a single manipulation. In the months leading up to an election, a company such as YouTube might be exposing users to similarly biased content repeatedly, and users themselves might choose to view certain videos multiple times, just as mass-murderer Brenton Harrison Tarrant did in Australia (see Introduction). If VME is an additive effect, multiple exposures will increase its impact, in which case the shifts we have produced in our experiments might be smaller than the shifts occurring in the natural environment. In ongoing experiments on what we call the “multiple exposure effect” (MEE) [160], we are now measuring the possible additive effects of VME, the “answer bot effect” (ABE) [5], the “search engine manipulation effect” (SEME) [2], and other new forms of influence made possible by the internet.

In the real world, bias in YouTube videos might also be similar to bias on other platforms, such as the Google search engine, Facebook, Instagram, and TikTok, as well as by answers given to users by AIs such as Bard and ChatGPT, in addition to answers given to users by intelligent personal assistants such as Alexa, the Google Assistant, and Siri. Again, in ongoing experiments, we are now measuring the possible additive effects of similarly biased content presented to users on different platforms, a phenomenon we called the “multiple platforms effect” (MPE).

The generalizability of our results is also limited by our subject pool (MTurk) and by the fact that our participants–all eligible US voters–did not have strong convictions about either the candidates or the issues in the election on which we focused (the 2019 election for Prime Minister of Australia). Our participants were not only undecided (as was confirmed by answers to our pre-manipulation questions), they were also “low information” (also sometimes called “low-familiarity”) voters, and low-information undecided voters can differ in nontrivial ways from high-information undecided voters [161]. These issues can only be addressed, we believe, by replicating our experiments with people who are more representative of actual voters than were the participants in our study. That said, we believe that VME poses little or no threat to people who have strong opinions about an issue or candidate; people who are undecided, on the other hand, are probably highly susceptible to VME and other new forms of influence that the internet has made possible.

In the real world, undecided voters are subject to many different kinds of influence when they are trying to make up their minds; YouTube is only one possible source of influence, needless to say, and some people rarely or never use YouTube. In addition, some people never use YouTube in a way that gives them information about political candidates or issues relevant to elections; in the US, in fact, the most common videos users searched for on YouTube in 2023 were BTS and Pewdiepie [162]. Our findings apply only to people who use YouTube fairly regularly and who are likely to encounter information relevant to elections.

That said, it is important to note here that the kind of influence YouTube can exert is especially powerful compared to other common sources of influence that affect people prior to elections. Most of those sources–television advertisements, billboards, and even ballot harvesting–are inherently competitive and often generate relatively small net effects, if any [163]. YouTube, on the other hand, has no effective competitor. Although opposing political campaigns can compete in the process of posting new video content to YouTube, only YouTube controls the process by which those videos are filtered, ordered, and customized. In other words, if employees, executives, or algorithms at YouTube favor one candidate, the opposing candidate has no way of counteracting that favoritism. Similarly, if employees, executives, or algorithms at YouTube choose to suppress the content of one candidate, that candidate has no way to counteract that suppression.

We note that our study and discussion have focused mainly on the potential that a YouTube-like platform has to alter people’s thinking and behavior. In our view YouTube presents at least two other major challenges to both researchers and policy makers, and we would be remiss in not pointing them out. First, YouTube has been repeatedly called to task in recent years for what some people consider to be censorship–removing content, restricting access to content, or demoting content on the platform [102, 104, 106–112]. We discussed this issue briefly in our Introduction, referring at one point to a PowerPoint presentation that leaked from Google entitled, “The Good Censor” [18]. Second, YouTube is a surveillance tool; Google openly tracks and subsequently models and monetizes the massive amount of data it collects about the videos people watch and the comments people post on YouTube [164, 165]. The information collected about children has been of special concern to authorities in recent years [166]. The present study needs to be viewed in this larger context. As a powerful and unprecedented tool of surveillance, censorship, and, it seems, manipulation, YouTube should, in our view, be subjected to close scrutiny by both researchers and public policy makers.

## Supporting information

S1 Text Candidate biographies. (DOCX)

S2 Text Instructions immediately preceding DoodleTube simulation. (DOCX)

S3 Text Vote Manipulation Power (VMP) calculation. (DOCX)

S1 Table Experiments 1&2: Demographics. (DOCX)

S2 Table Experiments 1&2: VMPs by gender. (DOCX)

S3 Table Experiments 1&2: VMPs by race/ethnicity. (DOCX)

S4 Table Experiments 1&2: VMPs by educational attainment. (DOCX)

S5 Table Experiments 1&2: Mean ratings on the 11-point scale of voting preference for the three groups by gender. (DOCX)

S6 Table Experiments 1&2: Mean ratings on the 11-point scale of voting preference for the bias groups (1&2) by gender. (DOCX)

S7 Table Experiments 1&2: Mean ratings on the 11-point scale of voting preference for the favored candidate by gender. (DOCX)

S8 Table Experiments 1&2: Mean ratings on the 11-point scale of voting preference for the three groups by educational attainment. (DOCX)

S9 Table Experiments 1&2: Mean ratings on the 11-point scale of voting preference for Groups 1&2 by educational attainment. (DOCX)

S10 Table Experiments 1&2: Mean preference for favored candidate on the 11-point scale of voting preference by educational attainment. (DOCX)

S11 Table Experiments 1&2: Mean ratings in the three groups on the 11-point scale of voting preference by race/ethnicity. (DOCX)

S12 Table Experiments 1&2: Mean preference shift for Groups 1&2 on the 11-point scale of voting preference by race/ethnicity. (DOCX)

S13 Table Experiments 1&2: Mean preference for favored candidate on the 11-point scale of voting preference by race/ethnicity. (DOCX)

S14 Table Experiment 1: Pre and Post opinion ratings of favored and non-favored candidates by race/ethnicity. (DOCX)

S15 Table Experiment 2: Pre and Post opinion ratings of favored and non-favored candidates by race/ethnicity. (DOCX)
