# Correction: The Video Manipulation Effect (VME): A quantification of the possible impact that the ordering of YouTube videos might have on opinions and voting preferences

**DOI:** 10.1371/journal.pone.0318389

**Published:** 2025-01-24

**Authors:** The PLOS One Staff

## Notice of Republication

This article was republished on January 16, 2025, to correct an error in the author list. Alex Flores was mistakenly omitted from the author byline. The publisher apologizes for this error. Please download the article again to view the corrected version. For reference, both the originally published, uncorrected article and the republished, corrected article are provided here.

## Supporting information

S1 File Originally published, uncorrected article. (PDF)

S2 File Republished, corrected article. (PDF)
