# Supplementary material for: The Video Manipulation Effect (VME): A quantification of the possible impact that the ordering of YouTube videos might have on opinions and voting preferences
Source: PLoS One. 2024 Nov 20;19(11):e0303036. doi: 10.1371/journal.pone.0303036 (PMC11578459; doi:10.1371/journal.pone.0303036)
Supplement: S2 Text — (DOCX) [file pone.0303036.s002.docx]

**S2 Text. Instructions immediately preceding DoodleTube simulation**

Thank you for your answers!

You will now be given an opportunity to learn more about these candidates using our special internet video platform called “DoodleTube.” Your goal is to try to clarify your views on the topic so you are better able to decide which candidate deserves your vote.

Use DoodleTube as you would normally use YouTube, and please do NOT use any other web pages to learn more about either candidate. In other words, please do not leave DoodleTube! If you do, that will invalidate your participation in our study. If you would like to conduct further research on the topic, go right ahead, but please complete our study first!

You will have a total of **15:00** minutes to view the videos, and the program will automatically let you know when the time is up.

Please do NOT close the window after conducting your search. Doing so will make it impossible for you to complete your participation in the study. Instead, if you feel you have enough information to make a clear choice, you may end your search early by clicking the “END” button in the upper-left corner of the DoodleTube page, which will appear after 10:00 minutes.

PLEASE NOTE: Some web pages might take a while to load, so please be patient.

Click the ‘Continue’ button below.
