# Supplementary material for: The Video Manipulation Effect (VME): A quantification of the possible impact that the ordering of YouTube videos might have on opinions and voting preferences
Source: PLoS One. 2024 Nov 20;19(11):e0303036. doi: 10.1371/journal.pone.0303036 (PMC11578459; doi:10.1371/journal.pone.0303036)
Supplement: S1 Table — (DOCX) [file pone.0303036.s004.docx]

|  | **Experiment 1**  **(*n* = 959)** | **Experiment 2**  **(*n* = 491)** |
| --- | --- | --- |
| **Mean Age (*SD*)** | 38.6 (12.0) | 36.4 (11.7) |
| **Gender (%)** |  |  |
| Female | 558 (58.2%) | 270 (55.0%) |
| Male | 391 (40.8%) | 218 (44.4%) |
| Unknown | 10 (1.0%) | 3 (0.6%) |
| **Education (%)** |  |  |
| None | 2 (0.2%) | 0 (0.0%) |
| Primary | 41 (4.3%) | 20 (4.1%) |
| Secondary | 309 (32.2%) | 160 (32.6%) |
| Bachelors | 428 (44.6%) | 224 (45.6%) |
| Masters | 148 (15.4%) | 72 (14.7%) |
| Doctorate | 31 (3.2%) | 15 (3.1%) |
| **Race/Ethnicity (%)** |  |  |
| White | 701 (73.1%) | 351 (71.5%) |
| Black | 102 (10.6%) | 55 (11.2%) |
| Asian | 75 (7.8%) | 39 (7.9%) |
| Mixed | 58 (6.0%) | 31 (6.3%) |
| Other | 22 (2.3%) | 15 (3.1%) |

**S1 Table. Experiments 1&2: Demographics**
