# Supplementary material for: The Video Manipulation Effect (VME): A quantification of the possible impact that the ordering of YouTube videos might have on opinions and voting preferences
Source: PLoS One. 2024 Nov 20;19(11):e0303036. doi: 10.1371/journal.pone.0303036 (PMC11578459; doi:10.1371/journal.pone.0303036)
Supplement: S2 Table — (DOCX) [file pone.0303036.s005.docx]

**S2 Table. Experiments 1&2: VMPs by gender.**

| Condition |  | *n* | VMP (%) | Bias (%) |
| --- | --- | --- | --- | --- |
| E1: No Mask | Male  Female | 268  376 | 40.6  58.9 | 33.6  32.4 |
|  | Change (%) | - | +45.1 | -3.6 |
|  | Statistic (*z*)  *p* | -  - | -4.58  < 0.001 | 0.32  0.749 NS |
| E2: Mask 2&3 | Male  Female | 154  180 | 52.6  77.9 | 14.3  15.0 |
|  | Change (%) | - | +48.1 | +4.9 |
|  | Statistic (*z*)  *p* | -  - | -4.87  < 0.001 | -0.18  0.857 NS |
