# Supplementary material for: The Video Manipulation Effect (VME): A quantification of the possible impact that the ordering of YouTube videos might have on opinions and voting preferences
Source: PLoS One. 2024 Nov 20;19(11):e0303036. doi: 10.1371/journal.pone.0303036 (PMC11578459; doi:10.1371/journal.pone.0303036)
Supplement: S3 Table — (DOCX) [file pone.0303036.s006.docx]

**S3 Table. Experiments 1&2: VMPs by race/ethnicity.**

| Condition |  | *n* | VMP (%) | Bias (%) |
| --- | --- | --- | --- | --- |
| E1: No Mask | White  Non-White | 477  174 | 51.9  50.5 | 34.8  28.2 |
|  | Change (%) | - | -2.7 | -19.0 |
|  | Statistic (*z*)  *p* | -  - | 0.32  0.749 NS | -1.58  0.114 NS |
| E2: Mask 2&3 | White  Non-White | 235  101 | 65.5  65.9 | 14.9  13.9 |
|  | Change (%) | - | +0.6 | -6.7 |
|  | Statistic (*z*)  *p* | -  - | -0.07  0.944 NS | 0.24  0.810 NS |
