# Supplementary material for: The Video Manipulation Effect (VME): A quantification of the possible impact that the ordering of YouTube videos might have on opinions and voting preferences
Source: PLoS One. 2024 Nov 20;19(11):e0303036. doi: 10.1371/journal.pone.0303036 (PMC11578459; doi:10.1371/journal.pone.0303036)
Supplement: S4 Table — (DOCX) [file pone.0303036.s007.docx]

**S4 Table. Experiments 1&2: VMPs by educational attainment.**

| Condition |  | *n* | VMP (%) | Bias (%) |
| --- | --- | --- | --- | --- |
| E1: No Mask | ≥ Bachelors  < Bachelors | 413  238 | 48.4  56.6 | 33.7  31.9 |
|  | Change (%) | - | +17.0 | -5.3 |
|  | Statistic (*z*)  *p* | -  - | -2.02  < 0.05 | 0.47  0.638 NS |
| Experiment 2: Mask 2&3 | ≥ Bachelors  < Bachelors | 224  112 | 63.7  72.5 | 15.6  12.5 |
|  | Change (%) | - | +13.8 | -19.9 |
|  | Statistic (*z*)  *p* | -  - | -1.61  0.107 NS | 0.76  0.447 NS |
