# Supplementary material for: The Video Manipulation Effect (VME): A quantification of the possible impact that the ordering of YouTube videos might have on opinions and voting preferences
Source: PLoS One. 2024 Nov 20;19(11):e0303036. doi: 10.1371/journal.pone.0303036 (PMC11578459; doi:10.1371/journal.pone.0303036)
Supplement: S5 Table — (DOCX) [file pone.0303036.s008.docx]

**S5 Table. Experiments 1&2: Mean ratings on the 11-point scale of voting preference for the three groups by gender.**

| **Condition** |  | ***n*** |  | ***M*_Morrison_ (SD)** | ***M*_Shorten_ (SD)** | ***M*_Control_ (SD)** | ***H*** | ***p*** |
| --- | --- | --- | --- | --- | --- | --- | --- | --- |
| E1: No Mask | Male | 391 | Pre | -0.31 (2.54) | -0.09 (2.79) | 0.01 (2.41) | 1.099 | 0.577 NS |
|  |  |  | Post | -1.06 (3.30) | 1.80 (3.31) | 0.32 (3.43) | 42.623 | < 0.001 |
|  | Female | 558 | Pre | -0.15 (2.90) | -0.16 (3.01) | 0.12 (3.01) | 0.991 | 0.609 NS |
|  |  |  | Post | -2.48 (2.95) | 2.35 (2.98) | 0.62 (3.58) | 152.927 | < 0.001 |
| E2: Mask 2&3 | Male | 218 | Pre | -0.44 (2.56) | -0.11 (2.74) | -0.69 (2.90) | 1.49 | 0.474 NS |
|  |  |  | Post | -1.57 (3.06) | 1.93 (3.13) | 0.31 (3.35) | 37.086 | < 0.001 |
|  | Female | 270 | Pre | 0.18 (3.04) | -0.19 (2.60) | 0.04 (2.86) | 0.764 | 0.682 NS |
|  |  |  | Post | -2.09 (3.37) | 2.09 (2.66) | 0.74 (3.66) | 56.572 | < 0.001 |
