# Supplementary material for: The Video Manipulation Effect (VME): A quantification of the possible impact that the ordering of YouTube videos might have on opinions and voting preferences
Source: PLoS One. 2024 Nov 20;19(11):e0303036. doi: 10.1371/journal.pone.0303036 (PMC11578459; doi:10.1371/journal.pone.0303036)
Supplement: S6 Table — (DOCX) [file pone.0303036.s009.docx]

**S6 Table. Experiments 1&2: Mean ratings on the 11-point scale of voting preference for the bias groups (1&2) by gender.**

| **Condition** |  | ***n*** | **Group 1 Shift** | **Group 2 Shift** |
| --- | --- | --- | --- | --- |
| E1: No Mask | Male | 268 | 0.75 | 1.89 |
|  | Female | 376 | 2.33 | 2.41 |
|  | Change (%) | - | +210.7 | +27.5 |
|  | *U* | - | 9860.500 | 11413.5 |
|  | *p* | - | < 0.001 | 0.371 NS |
| E2: Mask 2&3 | Male | 154 | 1.13 | 2.04 |
|  | Female | 180 | 2.27 | 2.28 |
|  | Change (%) | - | +100.9 | +11.8 |
|  | *U* | - | 3466 | 2859 |
|  | *p* | - | 0.089 NS | 0.834 NS |
