# Supplementary material for: The Video Manipulation Effect (VME): A quantification of the possible impact that the ordering of YouTube videos might have on opinions and voting preferences
Source: PLoS One. 2024 Nov 20;19(11):e0303036. doi: 10.1371/journal.pone.0303036 (PMC11578459; doi:10.1371/journal.pone.0303036)
Supplement: S7 Table — (DOCX) [file pone.0303036.s010.docx]

**S7 Table. Experiments 1&2: Mean ratings on the 11-point scale of voting preference for the favored candidate by gender.**

| **Condition** |  | ***n*** | ***M*_Pre_ (SD)** | ***M*_Post_ (SD)** | **Diff** | ***z*** | ***p*** |
| --- | --- | --- | --- | --- | --- | --- | --- |
| E1: No Mask | Male | 268 | 0.12 (2.67) | 1.41 (3.32) | 1.29 | -5.434 | < 0.001 |
|  | Female | 376 | -0.01 (2.96) | 2.41 (2.96) | 2.42 | -10.896 | < 0.001 |
|  | Change (%) | - | - | - | +87.6 | - | - |
|  | *Whitney U* | - | - | - | 42140.5 | - | - |
|  | *p* | - | - | - | < 0.001 | - | - |
| E2: Mask 2&3 | Male | 154 | 0.18 (2.65) | 1.74 (3.09) | 1.56 | -5.119 | < 0.001 |
|  | Female | 180 | -0.18 (2.84) | 2.09 (3.06) | 2.27 | -7.447 | < 0.001 |
|  | Change (%) | - | - | - | +45.5 | - | - |
|  | *U* | - | - | - | 12851.5 | - | - |
|  | *p* | - | - | - | 0.249 NS | - | - |
