# Supplementary material for: The Video Manipulation Effect (VME): A quantification of the possible impact that the ordering of YouTube videos might have on opinions and voting preferences
Source: PLoS One. 2024 Nov 20;19(11):e0303036. doi: 10.1371/journal.pone.0303036 (PMC11578459; doi:10.1371/journal.pone.0303036)
Supplement: S8 Table — (DOCX) [file pone.0303036.s011.docx]

**S8 Table. Experiments 1&2: Mean ratings on the 11-point scale of voting preference for the three groups by educational attainment.**

| **Condition** |  | ***n*** |  | ***M*_Morrison_ (SD)** | ***M*_Shorten_ (SD)** | ***M*_Control_ (SD)** | ***H*** | ***p*** |
| --- | --- | --- | --- | --- | --- | --- | --- | --- |
| E1: No Mask | ≥ Bachelors | 607 | Pre | -0.30 (2.68) | -0.10 (2.96) | 0.04 (2.67) | 1.531 | 0.465 NS |
|  |  |  | Post | -1.99 (3.12) | 2.18 (3.14) | 0.37 (3.47) | 131.140 | < 0.001 |
|  | < Bachelors | 352 | Pre | -0.09 (2.86) | -0.24 (2.80) | 0.05 (3.00) | 0.694 | 0.707 NS |
|  |  |  | Post | -1.63 (3.31) | 2.06 (3.05) | 0.61 (3.63) | 58.267 | < 0.001 |
| E2: Mask 2&3 | ≥ Bachelors | 311 | Pre | -0.05 (2.72) | -0.21 (2.69) | -0.39 (2.80) | 0.780 | 0.677 NS |
|  |  |  | Post | -1.53 (3.33) | 1.92 (3.00) | 0.29 (3.63) | 46.178 | < 0.001 |
|  | < Bachelors | 180 | Pre | -0.15 (3.11) | -0.04 (2.56) | -0.07 (2.99) | 0.055 | 0.973 NS |
|  |  |  | Post | -2.59 (2.93) | 2.25 (2.65) | 0.97 (3.38) | 54.058 | < 0.001 |
