# Supplementary material for: The Video Manipulation Effect (VME): A quantification of the possible impact that the ordering of YouTube videos might have on opinions and voting preferences
Source: PLoS One. 2024 Nov 20;19(11):e0303036. doi: 10.1371/journal.pone.0303036 (PMC11578459; doi:10.1371/journal.pone.0303036)
Supplement: S9 Table — (DOCX) [file pone.0303036.s012.docx]

**S9 Table. Experiments 1&2: Mean ratings on the 11-point scale of voting preference for Groups 1&2 by educational attainment.**

| **Condition** |  | ***n*** | **Group 1 Shift** | **Group 2 Shift** |
| --- | --- | --- | --- | --- |
| E1: No Mask | ≥ Bachelors | 413 | 1.69 | 2.28 |
|  | < Bachelors | 238 | 1.54 | 2.30 |
|  | Change (%) | - | -8.9 | +0.9 |
|  | *U* | - | 12434.5 | 11413.5 |
|  | *p* | - | 0.749 NS | 0.998 NS |
| E2: Mask 2&3 | ≥ Bachelors | 224 | 1.48 | 2.13 |
|  | < Bachelors | 112 | 2.44 | 2.29 |
|  | Change (%) | - | +64.9 | +7.5 |
|  | *U* | - | 3115 | 2563.5 |
|  | *p* | - | 0.120 NS | 0.666 NS |
