# Supplementary material for: The Video Manipulation Effect (VME): A quantification of the possible impact that the ordering of YouTube videos might have on opinions and voting preferences
Source: PLoS One. 2024 Nov 20;19(11):e0303036. doi: 10.1371/journal.pone.0303036 (PMC11578459; doi:10.1371/journal.pone.0303036)
Supplement: S10 Table — (DOCX) [file pone.0303036.s013.docx]

**S10 Table. Experiments 1&2: Mean preference for favored candidate on the 11-point scale of voting preference by educational attainment.**

| **Condition** |  | ***n*** | ***M*_Pre_ (SD)** | ***M*_Post_ (SD)** | **Diff** | ***z***^†^ | ***p*** |
| --- | --- | --- | --- | --- | --- | --- | --- |
| E1: No Mask | ≥ Bachelors | 413 | 0.09 (2.83) | 2.09 (3.13) | 2.00 | -9.836 | < 0.001 |
|  | < Bachelors | 238 | -0.07 (2.83) | 1.83 (3.19) | 1.90 | -6.897 | < 0.001 |
|  | Change (%) | - | - | - | -5.0 | - | - |
|  | *U* | - | - | - | 48484.5 | - | - |
|  | *P* |  |  |  | 0.773 NS |  |  |
| E2: Mask 2&3 | ≥ Bachelors | 224 | -0.07 (2.70) | 1.71 (3.18) | 1.78 | -6.744 | < 0.001 |
|  | < Bachelors | 112 | 0.06 (2.86) | 2.43 (2.80) | 2.37 | -6.064 | < 0.001 |
|  | Change (%) | - | - | - | +33.1 | - | - |
|  | *U* | - | - | - | 11331 | - | - |
|  | *p* | - | - | - | 0.146 NS | - | - |
