# Supplementary material for: The Video Manipulation Effect (VME): A quantification of the possible impact that the ordering of YouTube videos might have on opinions and voting preferences
Source: PLoS One. 2024 Nov 20;19(11):e0303036. doi: 10.1371/journal.pone.0303036 (PMC11578459; doi:10.1371/journal.pone.0303036)
Supplement: S11 Table — (DOCX) [file pone.0303036.s014.docx]

**S11 Table. Experiments 1&2: Mean ratings in the three groups on the 11-point scale of voting preference by race/ethnicity.**

| **Condition** |  | ***n*** |  | ***M*_Morrison_ (SD)** | ***M*_Shorten_ (SD)** | ***M*_Control_ (SD)** | ***H*** | ***p*** |
| --- | --- | --- | --- | --- | --- | --- | --- | --- |
| E1: No Mask | White | 701 | Pre | -0.30 (2.75) | -0.32 (2.92) | -0.05 (2.76) | 1.454 | 0.483 NS |
|  |  |  | Post | -1.90 (3.23) | 1.98 (3.14) | 0.51 (3.52) | 133.499 | < 0.001 |
|  | Non-White | 258 | Pre | -0.01 (2.75) | 0.35 (2.80) | 0.29 (2.88) | 0.793 | 0.673 NS |
|  |  |  | Post | -1.70 (3.09) | 2.61 (2.97) | 0.30 (3.57) | 56.318 | < 0.001 |
| E2: Mask 2&3 | White | 351 | Pre | -0.22 (2.82) | 0.06 (2.69) | -0.26 (2.86) | 0.729 | 0.695 NS |
|  |  |  | Post | -2.15 (3.05) | 2.11 (2.93) | 0.49 (3.55) | 78.349 | < 0.001 |
|  | Non-White | 140 | Pre | 0.27 (2.88) | -0.59 (2.52) | -0.23 (2.98) | 2.247 | 0.325 NS |
|  |  |  | Post | -1.19 (3.59) | 1.86 (2.79) | 0.87 (3.51) | 17.583 | < 0.001 |
