# Supplementary material for: The Video Manipulation Effect (VME): A quantification of the possible impact that the ordering of YouTube videos might have on opinions and voting preferences
Source: PLoS One. 2024 Nov 20;19(11):e0303036. doi: 10.1371/journal.pone.0303036 (PMC11578459; doi:10.1371/journal.pone.0303036)
Supplement: S12 Table — (DOCX) [file pone.0303036.s015.docx]

**S12 Table. Experiments 1&2: Mean preference shift for Groups 1&2 on the 11-point scale of voting preference by race/ethnicity.**

| **Condition** |  | ***n*** | **Group 1 Shift** | **Group 2 Shift** |
| --- | --- | --- | --- | --- |
| E1: No Mask | White | 477 | 1.60 | 2.30 |
|  | Non-White | 174 | 1.69 | 2.26 |
|  | Change (%) | - | +5.6 | -1.7 |
|  | *U* | - | 10594.5 | 10037 |
|  | *p* | - | 0.926 NS | 0.953 NS |
| E2: Mask 2&3 | White | 235 | 1.93 | 2.05 |
|  | Non-White | 101 | 0.92 | 1.27 |
|  | Change (%) | - | -52.3 | -38.0 |
|  | *U* | - | 3082.5 | 2563.5 |
|  | *p* | - | 0.351 NS | 0.858 NS |
