# Supplementary material for: The Video Manipulation Effect (VME): A quantification of the possible impact that the ordering of YouTube videos might have on opinions and voting preferences
Source: PLoS One. 2024 Nov 20;19(11):e0303036. doi: 10.1371/journal.pone.0303036 (PMC11578459; doi:10.1371/journal.pone.0303036)
Supplement: S13 Table — (DOCX) [file pone.0303036.s016.docx]

**S13 Table. Experiments 1&2: Mean preference for favored candidate on the 11-point scale of voting preference by race/ethnicity.**

| **Condition** |  | ***n*** | ***M*_Pre_ (SD)** | ***M*_Post_ (SD)** | **Diff** | ***z*** | ***p*** |
| --- | --- | --- | --- | --- | --- | --- | --- |
| E1: No Mask | White | 477 | -0.01 (2.85) | 2.94 (3.19) | 2.95 | -10.074 | < 0.001 |
|  | Non-White | 174 | 0.17 (2.77) | 2.14 (3.06) | 1.97 | -6.609 | < 0.001 |
|  | Change (%) | - | - | - | -34.2 | - | - |
|  | *U* | - | - | - | 41167 | - | - |
|  | *p* | - | - | - | 0.875 NS | - | - |
| E2: Mask 2&3 | White | 235 | 0.15 (2.76) | 2.13 (2.99) | 1.98 | -7.403 | < 0.001 |
|  | Non-White | 101 | -0.43 (2.71) | 1.51 (3.22) | 1.94 | -5.247 | < 0.001 |
|  | Change (%) | - | - | - | -2.02 | - | - |
|  | *U* | - | - | - | 11422.5 | - | - |
|  | *p* | - | - | - | 0.584 NS | - | - |
