# Supplementary material for: The Video Manipulation Effect (VME): A quantification of the possible impact that the ordering of YouTube videos might have on opinions and voting preferences
Source: PLoS One. 2024 Nov 20;19(11):e0303036. doi: 10.1371/journal.pone.0303036 (PMC11578459; doi:10.1371/journal.pone.0303036)
Supplement: S14 Table — (DOCX) [file pone.0303036.s017.docx]

**S14 Table. Experiment 1: Pre and Post opinion ratings of favored and non-favored candidates by race/ethnicity.**

| **Ethnicity** |  | **Favored Candidate Mean**  **(SD)** | | | **Non-Favored Candidate Mean (SD)** | | |  |
| --- | --- | --- | --- | --- | --- | --- | --- | --- |
|  |  | **Pre** | **Post** | **Diff** | **Pre** | **Post** | **Diff** | ***z***^†^ |
| White | Impression | 6.97 (1.88) | 7.37 (2.39) | 0.40 | 6.95 (1.94) | 4.64 (2.41) | -2.31 | -12.080*** |
|  | Trust | 6.16 (2.12) | 6.67 (2.57) | 0.51 | 6.22 (2.12) | 4.36 (2.36) | -1.86 | -11.181*** |
|  | Likeability | 6.91 (1.85) | 7.42 (2.47) | 0.51 | 6.96 (1.98) | 4.62 (2.49) | -2.34 | -12.647*** |
| Non-White | Impression | 7.36 (1.85) | 7.55 (2.24) | 0.19 | 7.17 (1.91) | 5.29 (2.49) | -1.88 | -6.336*** |
|  | Trust | 6.23 (1.93) | 6.80 (2.38) | 0.57 | 6.22 (1.94) | 4.97 (2.42) | -1.25 | -6.324*** |
|  | Likeability | 7.17 (1.84) | 7.46 (2.23) | 0.29 | 6.93 (1.90) | 5.31 (2.44) | -1.62 | -6.279*** |

^†^z-score represents Wilcoxon signed ranks test comparing post-minus-pre ratings for the favored candidate to the post-minus-pre ratings for the non-favored candidate

*** *p* < 0.001
