# Supplementary material for: The Video Manipulation Effect (VME): A quantification of the possible impact that the ordering of YouTube videos might have on opinions and voting preferences
Source: PLoS One. 2024 Nov 20;19(11):e0303036. doi: 10.1371/journal.pone.0303036 (PMC11578459; doi:10.1371/journal.pone.0303036)
Supplement: S15 Table — (DOCX) [file pone.0303036.s018.docx]

**S15 Table. Experiment 2: Pre and Post opinion ratings of favored and non-favored candidates by race/ethnicity.**

| **Ethnicity** |  | **Favored Candidate Mean (SD)** | | | **Non-Favored Candidate Mean (SD)** | | |  |
| --- | --- | --- | --- | --- | --- | --- | --- | --- |
|  |  | **Pre** | **Post** | **Diff** | **Pre** | **Post** | **Diff** | ***z***^†^ |
| White | Impression | 6.99 (1.70) | 7.43 (2.18) | 0.44 | 6.97 (1.74) | 4.89 (2.35) | -2.08 | -8.233*** |
|  | Trust | 6.26 (1.90) | 6.83 (2.24) | 0.57 | 6.17 (1.90) | 4.72 (2.39) | -1.45 | -7.429*** |
|  | Likeability | 6.94 (1.75) | 7.67 (2.12) | 0.75 | 6.92 (1.68) | 4.98 (2.44) | -1.94 | -8.549*** |
| Non-White | Impression | 6.73 (1.95) | 7.00 (2.20) | 0.27 | 6.94 (1.94) | 4.88 (2.43) | -2.06 | -5.343*** |
|  | Trust | 5.66 (2.20) | 6.38 (2.42) | 0.72 | 6.00 (2.31) | 4.47 (2.36) | -1.53 | -5.217*** |
|  | Likeability | 6.68 (1.93) | 7.08 (2.27) | 0.40 | 6.75 (2.00) | 5.01 (2.45) | -1.74 | -4.992*** |

^†^z-score represents Wilcoxon signed ranks test comparing post-minus-pre ratings for the favored candidate to the post-minus-pre ratings for the non-favored candidate

*** *p* < 0.001
