# Supplementary material for: Correction: The Video Manipulation Effect (VME): A quantification of the possible impact that the ordering of YouTube videos might have on opinions and voting preferences
Source: PLoS One. 2025 Jan 24;20(1):e0318389. doi: 10.1371/journal.pone.0318389 (PMC11760632; doi:10.1371/journal.pone.0318389)
Supplement: S1 File — (PDF) [file pone.0318389.s001.pdf]

PLOS ONE

RESEARCH ARTICLE

The Video Manipulation Effect (VME): A quantification of the possible impact that the ordering of YouTube videos might have on opinions and voting preferences

Robert Epstein*

American Institute for Behavioral Research and Technology, Vista, California, United States of America

* re@aibrt.org

Check for updates

OPEN ACCESS

Citation: Epstein R (2024) The Video Manipulation Effect (VME): A quantification of the possible impact that the ordering of YouTube videos might have on opinions and voting preferences. PLoS ONE 19(11): e0303036. https://doi.org/10.1371/journal.pone.0303036

Editor: Prabhat Mittal, Satyawati College (Eve.), University of Delhi, INDIA

Received: July 6, 2023

Accepted: April 15, 2024

Published: November 20, 2024

Copyright: © 2024 Robert Epstein. This is an open access article distributed under the terms of the Creative Commons Attribution License, which permits unrestricted use, distribution, and reproduction in any medium, provided the original author and source are credited.

Data Availability Statement: Anonymized raw data are accessible at https://zenodo.org/doi/10.5281/zenodo.11089923. Anonymization was required to comply with the requirement of the sponsoring institution's Institutional Review Board that the identities of the participants be protected in accordance with HHS Federal Regulation 45 CFR 46.101.(b)(2).

Funding: The authors received no specific funding for this work.

Abstract

Recent research has identified a number of powerful new forms of influence that the internet and related technologies have made possible. Randomized, controlled experiments have shown, for example, that when results generated by search engines are presented to undecided voters, if those search results favor one political candidate over another, the opinions and voting preferences of those voters can shift dramatically–by up to 80% in some demographic groups. The present study employed a YouTube simulator to identify and quantify another powerful form of influence that the internet has made possible, which we have labeled the Video Manipulation Effect (VME). In two randomized, controlled, counterbalanced, double-blind experiments with a total of 1,463 politically-diverse, eligible US voters, we show that when a sequence of videos displayed by the simulator is biased to favor one political candidate, and especially when the "up-next" video suggested by the simulator favors that candidate, both the opinions and voting preferences of undecided voters shift dramatically toward that candidate. Voting preferences shifted by between 51.5% and 65.6% overall, and by more than 75% in some demographic groups. We also tested a method for masking the bias in video sequences so that awareness of bias was greatly reduced. In 2018, a YouTube official revealed that 70% of the time people spend watching videos on the site, they are watching content that has been suggested by the company's recommender algorithms. If the findings in the present study largely apply to YouTube, this popular video platform might have unprecedented power to impact thinking and behavior worldwide.

1. Introduction

More than 5 billion people now have internet access, with these individuals potentially susceptible to efforts from tech companies to influence their thinking and behavior. Our research team has discovered and quantified several of these techniques in randomized, controlled experiments conducted since 2013. Our research demonstrates that some new types of

Competing interests: The authors have declared that no competing interests exist.

manipulation that the internet has made possible can easily shift votes and opinions without people's knowledge and without leaving paper trails for authorities to trace [1–7]. A growing body of non-scientific evidence suggests that Big Tech companies might sometimes use these new techniques of influence strategically and deliberately [8–12, cf. 13]. Below, we will give some examples of such evidence. In every instance, we acknowledge (a) that the industry professionals we quote are stating their opinions, (b) that we are relying in many instances on credible news sources, not academic journals, and (c) that the assertions made are not based on peer-reviewed research. We will leave it to the reader to decide how seriously to take this information. We offer it simply to put our present research into a larger social context.

1.2 Possible concerns about Big Tech influence

Tristan Harris, a former "design ethicist" at Google, has stated publically that he was a member of a team at the company whose job it was to influence "a billion people's attention and thoughts every day" [14]. Jaron Lanier, one of the early investors in Google and Facebook, claims that Big Tech content has "morphed into continuous behavior modification on a mass basis" [14]. Another early investor in these companies, Roger McNamee, has said that he now regrets having supported them, asserting that they now constitute "a menace to public health and to democracy" [15, cf. 14].

Three recent leaks of internal content from Google might also be cause for concern. First, in emails leaked from the company to *The Wall Street Journal* in 2018, employees were discussing how they might be able to change people's views about Trump's travel ban by using what they called "ephemeral experiences" [8]–that is, content such as search results and newsfeeds which appears briefly, impacts the user, and then disappears forever. Second, a 9-min video called "The Selfish Ledger" described the power that the company has to "sequence" human behavior "towards a desired result" according to "Google's values" [16, cf.17]. Third, "The Good Censor," a company PowerPoint presentation, explained that tech companies had been forced over the years to move away "from passive facilitation to active curation" of content, deciding what content users worldwide could and could not see [18].

In recent years, a number of authorities and experts have expressed particular concern about the way Google's YouTube platform might be influencing users, especially young children [19–26, cf. 27–36]. A 2019 *New York Times* investigation concluded that "YouTube's algorithms may have played a decisive role" in the rise of right-wing Brazilian president Jair Bolsonaro by "boost[ing] fringe videos into the mainstream" and helping to spread conspiracy theories and misinformation, especially about diseases [37]. In some cases, when users in Brazil were watching sports videos, YouTube's up-next suggestion (normally, the video image shown in the upper-right of the screen) would be for a Bolsonaro video, with one Bolsonaro video leading to others [29]. In some instances, just a few clicks have been known to take users down "rabbit holes" of similar videos making extreme claims that have sometimes radicalized them [20, 28, 29, 35–40, cf. 41–43]. This phenomenon has prompted sociologist Zeynep Tufekci to label YouTube as "one of the most powerful radicalizing instruments of the 21st century" [29, 34].

One of the most interesting cases of radicalization, reported in 2019, involved a 26-year-old White male named Caleb Cain of West Virginia–"a college dropout looking for direction" [28]. He turned to YouTube for guidance and was soon "pulled into a far-right filled universe, watching thousands of videos filled with conspiracy theories, misogyny and racism." He watched more than 12,000 such videos, falling, he later said, into "the alt-right rabbit hole." Cain's conversion apparently did not cause him to act violently, but other converts have been more aggressive. In 2020, Brenton Harrison Tarrant, a 28-year-old White male from Australia,

was convicted of 51 counts of murder, 40 counts of attempted murder, and one count of terrorism–the first terrorist convicted in New Zealand's history. His rampage took place in March, 2019, and his victims were worshippers at two mosques in Christchurch. Tarrant had been radicalized by YouTube videos. He even had made use of infinitelooper.com, which would repeat certain inspirational YouTube videos for him endlessly [44].

In 2022, the Anti-Defamation League published an ambitious study–well executed but not peer-reviewed, as far as we can tell–on user exposure to "alternative" and extremist content on YouTube [45]. Based on data obtained from a representative sample of 859 people in the US who were enrolled with YouGov, a national polling firm, the study concluded that roughly 1 in 5 YouTube users are exposed to "alternative" content–"channels that can serve as gateways to more extreme forms of content"–and that 1 in 10 users are exposed to extremist content directly. Although the researchers did not find evidence that extreme or disturbing YouTube content converted people with moderate views, they did find (a) that such content strongly attracted people "who already have high levels of racial resentment," (b) that when people watch such videos, they are "more likely to see and follow recommendations to similar videos," and (c) that when someone is viewing an extremist video, other extremist videos are likely to be recommended alongside them [45].

Some studies have found stronger evidence of radicalization on YouTube [46, 47, cf. 43, 48–53]. A large-scale 2020 study published by the Association for Computing Machinery that examined more than 330,000 videos concluded, for example, "We find strong evidence for radicalization among YouTube users, and that YouTube's recommender system enables Alt-right channels to be discovered, even in a scenario without personalization. . .. Moreover, regardless of the degree of influence of the recommender system in the process of radicalizing users, there is significant evidence that users are reaching content sponsoring fringe ideologies from the Alt-lite [people who 'flirt with' white supremacist ideology] and the Intellectual Dark Web" [46].

Other recent studies have catalogued and counted the growing number of extremist videos available on the YouTube platform [42, 46–50, 54–57]. Even though YouTube regularly removes many such videos from its platform, the number of alternative and extremist videos available to users worldwide on any given day is probably in the millions. This conjecture is based on recent surveys suggesting that upwards of 20% of internet users in the US have encountered hateful or harassing content on YouTube, which, at this writing (July 4, 2023), hosts more than 800 million videos [45, 58, 59]. The disturbing content could be in the videos themselves or in the comments provoked by those videos.

1.3 The value of controversial and addictive content

Both leaks and official statements from Big Tech platforms suggest that controversial content is an important part of content offerings because (a) it draws more traffic, and more traffic is generally more profitable [14, 34, 60, 61, cf. 35, 49], (b) it keeps people on a website longer [14, 35, 63, cf. 29, 37], and (c) it increases the "watch time" of videos [14, 35, cf. 29, 37]. Content personalization on platforms like YouTube has proved to be especially important in increasing the "stickiness" of websites [14, 28, 39, 62–64]. Even Mark Zuckerberg, the CEO of Facebook/Meta, acknowledged the value of controversial content from a business perspective in an official statement he released in 2018. According to Zuckerberg, "Our research suggests that no matter where we draw the lines for what is allowed, as a piece of content gets close to that line, people will engage with it more on average–even when they tell us afterwards they don't like the content" [65].

Multiple studies have also shown that YouTube's recommender algorithms are especially aggressive in recommending "pseudoscientific" videos and other content of dubious value [21,

22, 62, 66, cf. 67]; again, the more dubious the content, the more traffic is generated and the more watch time is increased. Anti-vaccine videos on YouTube have been shown to lead to recommendations of a disproportionately large number of additional anti-vaccine videos compared to pro-vaccine videos [68].

Even if radicalization on YouTube were rare, YouTube's video-management algorithm does allow it to occur. A vulnerable individual can be drawn into a highly persuasive sequence of videos when three important mechanisms are in alignment: filtering, ordering, and customization. Filtering is the process by which the algorithm selects some videos for presentation (a small sample) and rejects others (the vast majority). Ordering is the process by which the algorithm places one video ahead of another. And customization is the process by which the algorithm refines the filtering and ordering based on (a) information from the personal profile that Google has accumulated about the user and (b) priorities that the company or its employees might have about how they want to influence users. When these factors align, users can be caught in so-called "loops," "echo chambers," and "filter bubbles" of similarly biased content [48, 50, 69–78, cf. 79–83]. Relevant here is the fact that YouTube's algorithms also determine whether content goes viral on the platform [84, 85].

Note that all three of these factors also operate on Google's search engine. Although we tend not to think about YouTube this way, YouTube is actually the second largest search engine in the world, as well as the world's largest video-sharing social media platform [86]. The first video a user watches during a YouTube session is usually suggested after the user types a search term into YouTube's search bar. After that first search is completed, however, YouTube and the Google search engine part ways in how they influence the user. On Google, the user at some point clicks away to another website–ideally, from a business perspective, to a website Google wants the user to visit [4]. As Larry Page, co-founder of Google said famously long ago, "We want to get you out of Google and to the right place as fast as possible" [87]. Whether he meant the right place for the user or the right place for the company is unclear. On YouTube, the goal is the opposite: It is to keep the user on the platform as long as possible [14, 28, 29, 34, 35]. That behavioral addiction is the goal has been acknowledged by Google whistleblowers [14, 88, 89] and suggested by researchers [90, 91].

YouTube generally accomplishes these ends in ways that are too subtle for most users to discern. Users are likely aware that if they fail to search for another video or to click on one of the recommended videos (shown to the right of or just below the video screen), YouTube will automatically play another video–the up-next video shown on desktop and laptop computers in the upper-right corner of the computer screen. On smart phones, the up-next video will often play automatically even if the user has never seen the thumbnail version of it; this can occur, for example, when the phone is tilted to the landscape (horizontal) position, which causes the video that is playing to take up the entire screen. Until January 2015, a labeled "autoplay" on-off switch appeared above the up-next video on desktop and laptop computers which allowed the user to stop up-next videos from playing automatically. At this writing, however, the switch appears immediately below the video, and it no longer has a label on it; one must scroll over it (on desktop and laptop computers) or touch it (on mobile devices) even to find out what the button is for. These cosmetic changes were likely implemented to increase watch time [92–94, cf. 95–97]. To view images of YouTube's autoplay on-off switch pre- and post-January 2015, visit https://aibrt.org/downloads/VME_PLOS_2024/S1_Fig.jpg and https://aibrt.org/downloads/VME_PLOS_2024/S2_Fig.jpg.

According to official YouTube statements, watch time is YouTube's most important concern [61, 98, cf. 14]. In 2018, a YouTube official revealed that 70% of the time people spend watching videos on the site, they are watching content that has been suggested by the company's up-next algorithm [99]. The importance of watch time has also been emphasized in

public statements by former Google software engineer Guillaume Chaslot, who summarized this issue thus: "Watch time was the priority. Everything else was considered a distraction" [96]. When Chaslot suggested to his supervisors that the YouTube algorithm be modified to free users from content feedback loops, they rejected his ideas. "[T]he entire business model is based on watch time," according to Chaslot, and "divisive content" is especially effective in locking in user attention [14, cf. 39]. Tristan Harris expressed this concept metaphorically: "There's a spectrum on YouTube between the calm section–the Walter Cronkite, Carl Sagan part–and Crazytown, where the extreme stuff is. If I'm YouTube and I want you to watch more, I'm always going to steer you toward Crazytown" [28]. Unfortunately, such content can include "bizarre and disturbing" content directed at young children [100].

1.4 YouTube's power to influence users

According to various industry and news reports, YouTube exercises its power to influence and control by (a) demonetizing content it finds objectionable and thus discouraging certain content creators from posting videos [102–104, cf. 35, 101, 105], (b) restricting access to videos, in one case limiting access to more than 50 videos from the conservative Prager University organization, among them a video by noted Harvard Law School professor Alan Dershowitz about the founding of Israel and a video about the "thou shall not kill" provision of The Ten Commandments [106, 107, cf. 101], (c) deleting videos from its platform [108–112], and (d) reordering videos–in other words, boosting the positions of videos it is trying to promote and demoting videos it is trying to suppress [37, 57, 63, 64, 66]. In a 2-minute 2017 video leaked from Google in 2019 by a former Google staffer, Susan Wojcicki, then CEO of YouTube, explains to her staff the process by which YouTube's recommender algorithm was currently being altered to boost content the company viewed as valid and demote content the company considered suspect [113]. To view the full video, visit https://vimeo.com/354354050. That re-ranking process has continued to this day; at this writing (June 28, 2023), US Congressman and Presidential candidate Robert F. Kennedy Jr. is in the news protesting the removal of several of his videos from YouTube [114, 115].

When users have challenged such actions, US courts have repeatedly ruled in favor of Google and YouTube, asserting that by deleting or reordering content, these platforms, as private companies, are exercising their right to free speech under the First Amendment to the US Constitution [106, 116–119].

A growing body of research demonstrates the power of YouTube's recommender algorithms, either to cause people to formulate opinions where their opinions are initially weak, or to further strengthen opinions where opinions are initially strong [66, 71–74, cf. 78, 120–122]. Some of this research extends these general findings to the political realm [123]. For example, a 2020 study by Cho et al. (conducted with 108 undergraduate students at one university) demonstrated the power that YouTube's recommender algorithms have to "reinforce and polarize" existing political opinions [76, cf. 77]. Newer studies suggest that YouTube's up-next algorithm might be biased to some extent in one direction politically [43, 81], although "communities" of YouTube users can have almost any political bias [50, 120].

Whether videos are generally more influential than auditory, textual, or still-image media is a matter that has not been well explored, to our knowledge–in part, we believe, because of the difficulties inherent in designing studies that compare the persuasiveness of these media fairly. One recent study suggests, however, that video is substantially more powerful than text in convincing people that political content is real, but that it is only slightly more persuasive than text [124, 125, cf. 126–131]. Whatever the truth is about direct comparisons, researchers have consistently found that videos get far more "shares" online than other forms of media do–

according to one recent estimate, "1200% more shares than text and images combined" [132, cf. 133], and online content apparently has far more impact, in general, than offline content [134, cf. 123].

1.5 The importance of capturing ephemeral content

These new methods of influence are especially problematic because they are controlled worldwide (outside the People's Republic of China) by a small number of corporate monopolies, which means one cannot counteract them. If a political candidate airs an attack ad on television or on the internet, the opponent can air a rejoinder. But if a large online platform uses new techniques of influence to support a candidate, the opponent can do nothing to counteract that influence; in many cases, that manipulation might not even be visible. As we noted earlier, many online manipulations also make strategic use of ephemeral experiences to change thinking or behavior; that normally guarantees that these manipulations leave no paper trails for authorities to trace. Note that although YouTube videos are *not* ephemeral, the video sequences and up-next suggestions the company makes are indeed ephemeral. They are generated on the fly for the individual user and stored nowhere, and there is no way, to our knowledge, for anyone–including Google employees–to go back in time to regenerate them.

We and our colleagues have successfully built monitoring systems that have preserved increasingly larger bodies of ephemeral experiences in the days leading up to six elections in the US [135–137]. In 2020, we preserved and analyzed more than 1.5 million ephemeral experiences obtained and then aggregated through the computers of a politically-diverse group of 1,735 registered voters in four swing states. We captured data on the Google, Bing, and Yahoo search engines, as well as on YouTube and Google's home page [138, 139], and we found substantial political bias on these platforms, sufficient, perhaps, to have shifted millions of votes among undecided voters. Based on our preliminary analysis of data we had collected, on November 5, 2020, three US Senators sent a warning letter to the CEO of Google about the political bias we had detected in Google content, and Google immediately turned off political bias in the search results it was sending to Georgia residents in the weeks leading up to the two US Senate runoff elections scheduled there for January 5, 2021. Google also stopped sending go-vote reminders to Georgia residents. Monitoring systems, it appears, can be used to make Big Tech companies accountable to the public. As Supreme Court Justice Louis D. Brandeis opined a century ago, "Sunlight is said to be the best of disinfectants; electric light the most efficient policeman" [140].

In 2022, we expanded our network of "field agents" to include 2,742 registered voters, and we preserved more than 2.5 million ephemeral experiences on multiple platforms, this time including both Twitter and Facebook [141]. We are currently in the process of building a permanent, large-scale "digital shield" in all 50 US states which will, we hope, protect our elections from manipulation by emerging technologies for the foreseeable future [141–143]. At this writing (March 20, 2024), this automated system is preserving and analyzing ephemeral online content 24 hours a day from the computers of a politically-balanced group of more than 14,000 registered voters in all 50 US states [144], and we have thus far preserved more than 80 million ephemeral experiences.

Proposals have also been made to try to track or reduce the potential manipulative power of software such as YouTube's recommender algorithms by developing methods that increase algorithmic transparency and accountability [145–149, cf. 150]. The companies that control these algorithms will likely resist such efforts, however, and because of their increasing reliance on machine learning techniques, algorithms have grown increasingly opaque over the years–so mysterious that even the original programmers can't understand them [151]. The clearest

way, in our view, to monitor, preserve, and analyze algorithmic output is to look over the shoulders of large, representative samples of real users as they are viewing real content. Doing so is necessary in part because so much content is now customized to fit characteristics of individual users [152–154].

1.6 Video Manipulation Effect (VME)

The present paper focuses on a powerful new form of influence we call the Video Manipulation Effect (VME), in which we use a YouTube simulator we call DoodleTube to determine the extent to which we can shift the opinions and voting preferences of undecided voters by manipulating the order of recommended videos–in other words, by exercising control over YouTube's recommender algorithms. The videos were biased to favor one candidate or his opponent. By manipulating the order, we also had control over which video was in the up-next position and which therefore would play automatically if the user did not select a different video. By parsing the data demographically, we also determined how vulnerable people in different demographic groups were to the manipulation.

2. Experiment 1: Biased video ordering with no mask

In our first experiment, we sought to determine whether a biased ordering of recommended videos–biased to favor one political candidate–could shift opinions and voting preferences toward that candidate. By "no mask," we mean that high-ranking videos consistently favored one candidate. In Experiment 2, in order to reduce perception of bias, we masked the bias by mixing in videos that supported the non-favored candidate (see Procedure sections below for details).

2.1 Methods

2.1.1 Ethics statement. The federally registered Institutional Review Board (IRB) of the sponsoring institution (American Institute for Behavioral Research and Technology) approved this study with exempt status under US Health and Human Services (HHS) rules because (a) the anonymity of participants was preserved and (b) the risk to participants was minimal. AIBRT is registered with the HHS Office for Human Research Protections (OHRP) under IORG0007755. The IRB is registered with OHRP under number IRB00009303, and the Federalwide Assurance number for the IRB is FWA00021545. Informed written consent was obtained for both experiments as specified in the Procedure section of Experiment 1.

2.1.2 Participants. After cleaning, our participant sample for this experiment consisted of 959 eligible US voters recruited through the Amazon Mechanical Turk (MTurk) subject pool. During the cleaning process, we removed participants who reported an English fluency level below 6 on a 10-point scale, where 1 was labeled "Not fluent" and 10 was labeled "Highly fluent." In order to assure that our participants were undecided, we also removed participants who reported a level of familiarity with either of the two candidates exceeding 3 on a 10-point scale. In all, 41 participants were removed during cleaning.

Overall, 558 (58.2%) of our participants identified themselves as female, 391 (40.8%) as male, and 10 (1.0%) chose not to identify their gender. Racial and ethnic background was as follows: 701 (73.1%) of our participants identified themselves as White, 102 (10.6%) as Black, 75 (7.8%) as Asian, 58 (6.0%) as Mixed, and 22 (2.3%) as Other. Overall, 26.8% of the individuals in the sample identified themselves as non-White.

Regarding level of education completed: 2 (0.2%) of our participants reported no education; 41 (4.3%) reported not having a high school degree; 309 (32.2%) reported completing high

school; 428 (44.6%) reported having a bachelor's degree; 148 (15.4%) reported having a master's degree; and 31 (3.2%) reported having a doctoral degree.

Regarding political alignment: 435 (45.4%) of our participants identified themselves as liberal, 291 (30.3%) as moderate, 187 (19.5%) as conservative, 30 (3.1%) as not political, and 16 (1.7%) as other.

Regarding YouTube usage: 958 (99.9%) of our participants reported that they have used YouTube before, and 602 (62.8%) reported that they have used YouTube to get information about political or ideological topics. Participants reported using YouTube an average of 18.0 (SD = 32.4) times a week.

See S1 Table for detailed demographic information for Experiment 1.

2.1.3 Procedure. All procedures were conducted online, with sessions conducted on December 7, 2021, December 11, 2021, and January 7, 2022. Participants were first asked two screening questions; sessions were terminated if they said they were not eligible to vote in the US or if they reported a level of familiarity with Australian politics exceeding 3 on a 10-point scale. We chose to exclude people who were highly familiar with Australian politics in order to assure that our participants–all from the US–were likely to be undecided about which candidate to vote for in the election we featured in the experiment: the 2019 election for Prime Minister of Australia.

Participants who passed our screening questions were then asked various demographic questions and then given instructions about the experimental procedure. We also displayed a short video and asked participants whether they were able to see the video and whether the video autoplayed. If the videos did not play at all or did not autoplay the session was terminated. At the end of the instructions page, and in compliance with American Psychological Association and HHS guidelines, participants were asked for their consent to participate in the study. If they clicked "I Do," the session continued; if they clicked "I Do Not," the session ended. Participants were then asked further questions about their political leanings and voting behavior.

Participants were then given a short paragraph about each of two candidates who ran for Prime Minister of Australia in 2019, each about 120 words in length (see S1 Text in Supporting Information for the full paragraphs). Participants were next asked three opinion questions on a 10-point scale about each candidate: one regarding their overall impression of the candidate, one regarding how likeable they found the candidate, and one regarding how much they trusted the candidate. They were then asked, on an 11-point scale with values ranging from 5 to 0 to 5, which candidate they would be likely to vote for if they "had to vote today." Finally, they were asked which candidate they would vote for if they "had to vote right now" (forced choice).

Participants were then given an opportunity to use DoodleTube–our YouTube simulator–to watch videos about these candidates in order to gather information to help them decide which of the two candidates to vote for. They were given a maximum of 15 minutes and a minimum time of 10 minutes to view the videos. See S2 Text for the complete instructions.

On the next screen, participants saw an online video platform called DoodleTube displaying a search bar with a pre-inputted query of "Australian Prime Minister Election" and a series of videos relating to that query (Fig 1). Participants could click on any of the videos to play it. When a video was clicked, the screen switched to a video view screen with the up-next video on the top of the right side bar, along with other recommended videos beneath it (Fig 2).

The participant could watch an entire video, or the participant could click on a different video to switch the view to that new one. The participant could also allow a video to play to the end and then allow the up-next video to play; this occurred automatically if the participants did not click on another video.

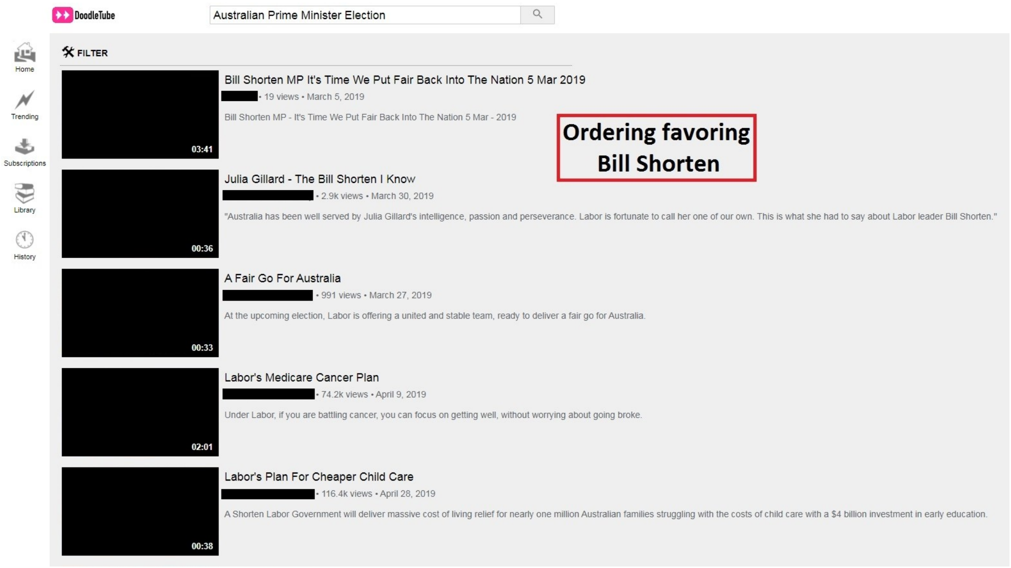

Fig 1. Initial screen when a DoodleTube session begins, with identifying images and information removed. In this instance, the participant had been randomly assigned to a group in which the order of the videos favored candidate Bill Shorten. The red-outline box above was not shown. To view the unblocked image, visit https://aibrt.org/downloads/VME_PLOS_2024/Fig1.jpg.

https://doi.org/10.1371/journal.pone.0303036.g001

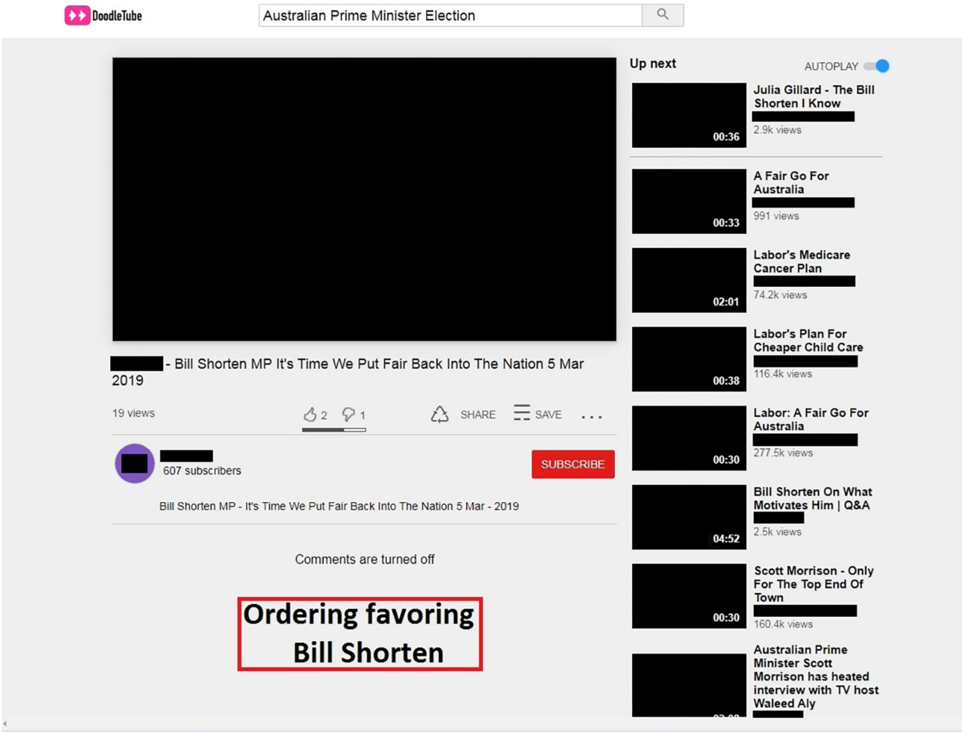

Fig 2. Screen that appears after the participant has clicked one of the videos shown in Fig 1, with identifying images and information removed. The red-outlined box above was not shown. To view the unblocked image, visit https://aibrt.org/downloads/VME_PLOS_2024/Fig2.jpg.

https://doi.org/10.1371/journal.pone.0303036.g002

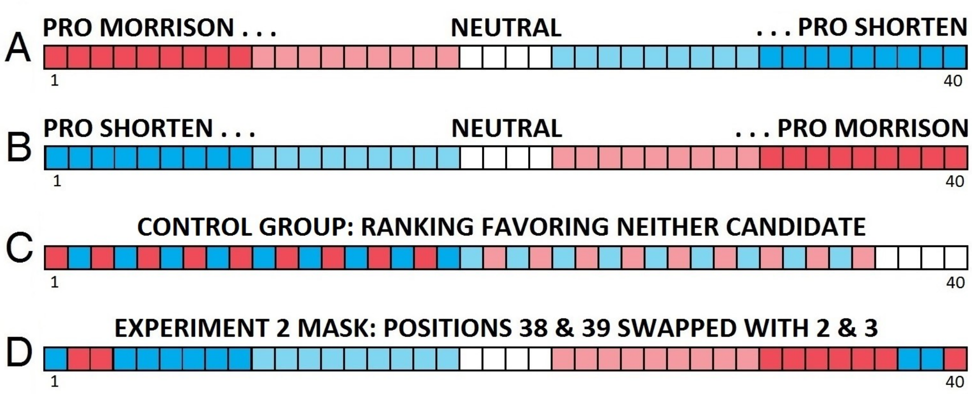

Fig 3. Ordering of videos in Experiments 1 and 2. A: Experiment 1, Group 1 (first bias group, 40 videos in order from Pro Morrison to neutral to Pro Shorten). B: Experiment 1, Group 2 (second bias group, 40 videos in order from Pro Shorten to neutral to Pro Morrison). C: Group 3 (control group, 40 videos in which Pro Morrison and Pro Shorten videos are alternated). D: Experiment 2: The manipulation is masked by swapping the video in Position 2 with the video in Position 39, and the video in Position 3 with the video in Position 38 (shown only for Group 1, Pro Morrison bias group).

https://doi.org/10.1371/journal.pone.0303036.g003

Participants had been randomly assigned to one of three groups: Pro-Candidate-A (Scott Morrison), Pro-Candidate-B (Bill Shorten), or the control group. People in all three groups had access to all 40 of the videos that were included in the experiment, but the videos were listed in a different order in each group. As shown in Fig 3A, in the Pro-Morrison group, the order of the videos would go from Pro-Morrison videos to Pro-Shorten videos. If the participant was assigned to the Pro-Shorten group, the order of the videos would go from Pro-Shorten videos to Pro-Morrison videos (Fig 3B). The control group would have both Pro-Morrison and Pro-Shorten videos in alternation (Fig 3C).

The End button in the upper-left corner of the web page was invisible and inaccessible for the minimum required video view time of 10 minutes. After the 10 minutes was up, the End button appeared. However, participants were allowed to continue browsing the videos up to the maximum time of 15 minutes. When the 15 minutes was up, the participants were redirected to the questions page. The timer was paused when the videos were paused and restarted when the videos were restarted to ensure participants were viewing the video for the required amount of time. The autoplay feature was on by default and could not be turned off. Users could, however, go to whatever video they desired either on the sidebar or the home screen.

Following the DoodleTube experience, participants were again asked the same series of questions that they were asked before they began watching the videos: three opinion questions about each candidate (10-point scale on impression, likeability, and trust), which candidate they were likely to vote for (11-point scale from 5 to 0 to 5), and which candidate they would vote for now (forced choice).

Next, participants were asked whether any of the content they had seen on DoodleTube "bothered" them in any way. They could reply Yes or No, and then they could explain their answer by typing freely in a text box. This is a conservative way of determining whether people perceived any bias in the content they had seen. We could not ask people directly about their awareness of bias because leading questions of that sort often produce misleading answers [155]. To assess bias we searched the textual responses for words such as "bias," "skewed," or "slanted" to identify people in the bias groups who had apparently noticed the favoritism in the search results they had been shown.

2.2 Results

For election campaign officials, the most important result in this experiment would almost certainly be what we call the "vote manipulation power" or VMP, which we define as the post-manipulation percentage increase in the number of participants preferring the favored candidate (in response to the forced-choice question). In a group that is initially split 50/50, the VMP also turns out to be the post-manipulation vote margin. For additional information about VMP and how we compute it, see S3 Text in the Supporting Information.

In Experiment 1, for all participants in the two bias groups combined (Groups 1 and 2), the VMP was 51.5% (McNemar's Test $X^2 = 98.20$, $p < 0.001$). S2–S4 Tables show VMPs broken down by gender, race/ethnicity, and level of educational attainment. For those demographic characteristics, we found significant and consistent effects only for gender, with females having higher VMPs than males.

On the 11-point voting preferences scale, pre-manipulation, we found no significant difference between mean ratings in the three groups ($M_{\text{Morrison}} = -0.22$, $SD = 2.75$; $M_{\text{Shorten}} = -0.15$, $SD = 2.90$; $M_{\text{Control}} = 0.04$, $SD = 2.81$; Kruskal-Wallis $H = 1.53$; $p = 0.47$ NS). Post manipulation, we found a significant difference between mean ratings in the three groups ($M_{\text{Morrison}} = -1.85$, $SD = 3.19$; $M_{\text{Shorten}} = 2.14$, $SD = 3.11$; $M_{\text{Control}} = 0.45$, $SD = 3.53$; $H = 188.67$; $p < 0.001$). Participants in Group 1 shifted 1.63 points towards the favored candidate (Morrison), and participants in the Group 2 condition shifted 2.29 points towards the favored candidate (Shorten). In addition, the pre-manipulation mean preference for the favored candidate (Groups 1 and 2 combined) was significantly different from the post-manipulation mean preference for the favored candidate (Groups 1 and 2 combined) ($M_{\text{Pre}} = 0.04$, $SD_{\text{Pre}} = 2.83$, $M_{\text{Post}} = 1.99$, $SD_{\text{Post}} = 3.15$, Wilcoxon $z = -12.02$, $p < 0.001$). Opinion ratings for both candidates also shifted significantly in the predicted direction (Table 1). Finally, the proportion of videos our participants watched which were selected by our up-next recommendation was 56.8% ($SD = 27.1$). (Non-parametric statistical tests such as the Kruskal-Wallis H are frequently employed in this study because the ratings of the candidates lie on ordinal scales [156]. Means and standard deviations in such instances are reported for comparison purposes, although the appropriateness of their use with ordinal data has long been debated [157, 158]).

Demographic breakdowns of the data obtained on the 11-point voting preference scale in Experiment 1 are shown in S5–S13 Tables. Male/female differences on this scale were highly significant (S5–S7 Tables). Differences by educational attainment and race/ethnicity were not consistently significant (S8–S13 Tables).

Although the shift in voting preferences was substantial in Experiment 1 (VMP = 51.5%), it is notable that 33.0% of the participants in the two bias groups (Groups 1 and 2) appeared to detect political bias in the videos they watched. In SEME experiments, perception of bias can easily be reduced with masking procedures–for example, by mixing one or more search results favoring the non-favored candidate mixed among the more frequent and higher-ranking

Table 1. Experiment 1: Pre and post opinion ratings of favored and non-favored candidates.

	Favored Candidate Mean (SD)			Non-Favored Candidate Mean (SD)			
	Pre	Post	Diff	Pre	Post	Diff	$z^{\dagger}$
Impression	7.07 (1.87)	7.42 (2.35)	0.35	7.01 (1.93)	4.82 (2.45)	-2.19	-13.64***
Trust	6.18 (2.07)	6.70 (2.52)	0.52	6.22 (2.07)	4.52 (2.39)	-1.70	-12.84***
Likeability	6.98 (1.85)	7.43 (2.41)	0.45	6.95 (1.96)	4.81 (2.50)	-2.14	-14.12***

†z-score represents Wilcoxon signed ranks test comparing post-minus-pre ratings for the favored candidate to the post-minus-pre ratings for the non-favored candidate

*** $p < 0.001$

https://doi.org/10.1371/journal.pone.0303036.t001

search results favoring the other candidate [2]. Could we reduce perception of bias in a YouTube-like environment using a similar mask, and, if so, might we still be able to produce a substantial shift in voting preferences? We attempted to answer these questions in Experiment 2.

3. Experiment 2: Biased video ordering with mask

3.1 Methods

3.1.1 Participants. After cleaning, our participant sample consisted of 491 eligible US voters recruited through the MTurk subject pool. The cleaning procedure was identical to that of Experiment 1, and a total of nine participants were removed from the sample during that procedure. The group was demographically diverse. See S1 Table for detailed demographic information for Experiment 2.

Regarding YouTube usage: 489 (99.6%) reported that they had used YouTube before, and 310 (63.1%) reported that they had used YouTube to get information about political or ideological topics. Participants reported using YouTube an average of 16.2 (SD = 30.2) times per week.

3.1.2 Procedure. The procedure in Experiment 2, with sessions conducted on December 24, 2021, and January 8, 2022, was identical to that of Experiment 1, with one exception: The order of recommended videos in the experimental groups had a mask in the 2nd and 3rd positions. Specifically, we swapped the usual video in Position 2 with the video from Position 39, and the usual video in Position 3 with the video from Position 38 (see Fig 3D). In other words, in the Pro-Morrison group, the video order remained the same as it was in Experiment 1 except in the 2nd and 3rd positions the videos were Pro-Shorten. Similarly for Pro-Shorten group, the videos in the 2nd and 3rd positions were Pro-Morrison.

3.2 Results

In Experiment 1 (no mask), the VMP was 51.5%, and 33.0% of participants in the two bias groups showed some awareness of bias in the ordering of the videos. In Experiment 2 (mask), the VMP was 65.6% (McNemar's Test X^2 = 67.11, $p < 0.001$), and only 14.6% of participants in the two bias groups showed some awareness of bias in the ordering of the videos. The VMP in Experiment 2 was 27.4% higher than the VMP in Experiment 1 (z = 4.23, $p < 0.001$). The perception of bias in Experiment 2 was 55.8% lower than the perception of bias in Experiment 1 (z = 6.19, $p < 0.001$). Thus it appears that the manipulation can indeed be masked in such a way as to reduce perception of bias (perhaps to zero) while still producing a substantial shift in voting preferences. Again, S2–S4 Tables show VMPs broken down by gender, race/ethnicity, and level of educational attainment. For those demographic characteristics, we again found consistently significant effects only for gender, with females having higher VMPs than males.

Voting preferences as measured on the 11-point scale also shifted in the predicted direction. Pre-manipulation, we found no significant difference between mean ratings in the three groups ($M_{Morrison}$ = -0.08, SD = 2.84; $M_{Shorten}$ = -0.15, SD = 2.65; $M_{Control}$ = -0.25, SD = 2.88; H = 0.27, p = 0.873 NS). Post manipulation, we found a significant difference between mean ratings in the three groups ($M_{Morrison}$ = -1.87, SD = 3.23; $M_{Shorten}$ = 2.03, SD = 2.88; $M_{Control}$ = 0.59, SD = 3.53; H = 95.64; $p < 0.001$). Participants in Group 1 shifted 1.79 points towards the favored candidate (Morrison), and participants in the Group 2 condition shifted 2.18 points towards the favored candidate (Shorten). In addition, the pre-manipulation mean preference for the favored candidate (Groups 1 and 2 combined) was significantly different from the post-manipulation mean preference for the favored candidate (Groups 1 and 2 combined) (M_{Pre} = -0.02, SD_{Pre} = 2.75; M_{Post} = 1.95, SD_{Post} = 3.07; Wilcoxon z = -9.05, $p < 0.001$). Opinion ratings for both candidates also shifted significantly in the predicted direction (Table 2).

Table 2. Experiment 2: Pre and post opinion ratings of favored and non-favored candidates.

	Favored Candidate Mean (SD)			Non-Favored Candidate Mean (SD)			
	Pre	Post	Diff	Pre	Post	Diff	$z^{\dagger}$
Impression	6.91 (1.78)	7.30 (2.22)	0.39	6.96 (1.80)	4.89 (2.37)	-2.07	-9.81***
Trust	6.08 (2.01)	6.69 (2.30)	0.61	6.12 (2.03)	4.65 (2.38)	-1.47	-9.06***
Likeability	6.86 (1.81)	7.49 (2.18)	0.63	6.87 (1.79)	4.99 (2.44)	-1.88	-9.91***

† z-score represents Wilcoxon signed ranks test comparing post-minus-pre ratings for the favored candidate to the post-minus-pre ratings for the non-favored candidate

*** $p < 0.001$

https://doi.org/10.1371/journal.pone.0303036.t002

Finally, the proportion of videos our participants watched which were selected by our up-next recommendation was 60.7% (*SD* = 25.6).

Demographic breakdowns of the data obtained on the 11-point voting preference scale in Experiment 2 are shown in S5–S13 Tables. Male/female differences on this scale were not consistently significant (S8–S13 Tables). Neither were differences by educational attainment or race/ethnicity (S8–S13 Tables).

4. Discussion

Our experiments demonstrate that (a) strategic ordering of videos on a YouTube-like platform can dramatically shift both the opinions and voting preferences of undecided voters, rapidly shifting a substantial portion of them to favor one political candidate (Experiment 1), and (b) this manipulation can be masked to reduce perception of bias while still producing large, predictable shifts in opinions and voting preferences (Experiment 2). These findings are important because, as we have documented in our Introduction, an increasing body of evidence suggests that YouTube itself can have a dramatic impact on the thinking and behavior of people worldwide, sometimes in destructive or self-destructive ways.

Like search results, search suggestions, answer boxes, and newsfeeds, video sequences constructed by YouTube's recommender algorithm are ephemeral in nature. Once again, that means that this form of influence normally leaves no paper trail for authorities to trace or, perforce, for researchers to study. Without monitoring systems in place to preserve large representative samples of ephemeral content, people will be blind to the ways in which they are being impacted by the algorithms of tech companies, and we will in effect be turning our democracy, and, to some extent, our own minds over to these companies. Some children are especially impressionable [32, 33, 159], and with mobile devices having now become both the babysitter and the companion of choice for children [19, 23], it is reasonable to conjecture that the new forms of influence that the internet has made possible are impacting our children and grandchildren profoundly [20–22, 24–27, 31]. In our view, laws and regulations will never be able to keep pace with rapidly changing technologies, but monitoring systems can. It's good tech battling bad tech, now and in the future.

In recent months, our team has preserved more than 70 million online ephemeral experiences in all 50 US states through the computers of a politically-balanced group of more than 13,000 registered voters, and we are now beginning to preserve content from the mobile devices of more than 2,000 children and teens (with the permission of their parents). We are also developing ways of analyzing much of this data in real time, and we recently began giving both the authorities and the general public free access to our findings 24 hours a day through a public dashboard [145]. In our view, in a world in which unprecedented power has been given to private companies to impact people's thinking and behavior, monitoring systems are not optional. Without them, we will not only have no idea how such companies might be

influencing us, governments that implement laws or regulations to contain the power of these companies will have no reliable way of measuring compliance with those laws and regulations.

4.1 Limitations and future research

Our current procedures do not include any follow up, so we have no way of measuring how long the changes our procedures produce in opinions and voting preferences will last. Just as the content our participants see is fleeting, so might be the changes we are detecting in their opinions and voting preferences. That said, we might actually be underestimating the possible power of VME as it might be impacting real users, because we are exposing our participants to only a single manipulation. In the months leading up to an election, a company such as YouTube might be exposing users to similarly biased content repeatedly, and users themselves might choose to view certain videos multiple times, just as mass-murderer Brenton Harrison Tarrant did in Australia (see Introduction). If VME is an additive effect, multiple exposures will increase its impact, in which case the shifts we have produced in our experiments might be smaller than the shifts occurring in the natural environment. In ongoing experiments on what we call the "multiple exposure effect" (MEE) [160], we are now measuring the possible additive effects of VME, the "answer bot effect" (ABE) [5], the "search engine manipulation effect" (SEME) [2], and other new forms of influence made possible by the internet.

In the real world, bias in YouTube videos might also be similar to bias on other platforms, such as the Google search engine, Facebook, Instagram, and TikTok, as well as by answers given to users by AIs such as Bard and ChatGPT, in addition to answers given to users by intelligent personal assistants such as Alexa, the Google Assistant, and Siri. Again, in ongoing experiments, we are now measuring the possible additive effects of similarly biased content presented to users on different platforms, a phenomenon we called the "multiple platforms effect" (MPE).

The generalizability of our results is also limited by our subject pool (MTurk) and by the fact that our participants–all eligible US voters–did not have strong convictions about either the candidates or the issues in the election on which we focused (the 2019 election for Prime Minister of Australia). Our participants were not only undecided (as was confirmed by answers to our pre-manipulation questions), they were also "low information" (also sometimes called "low-familiarity") voters, and low-information undecided voters can differ in nontrivial ways from high-information undecided voters [161]. These issues can only be addressed, we believe, by replicating our experiments with people who are more representative of actual voters than were the participants in our study. That said, we believe that VME poses little or no threat to people who have strong opinions about an issue or candidate; people who are undecided, on the other hand, are probably highly susceptible to VME and other new forms of influence that the internet has made possible.

In the real world, undecided voters are subject to many different kinds of influence when they are trying to make up their minds; YouTube is only one possible source of influence, needless to say, and some people rarely or never use YouTube. In addition, some people never use YouTube in a way that gives them information about political candidates or issues relevant to elections; in the US, in fact, the most common videos users searched for on YouTube in 2023 were BTS and Pewdiepie [162]. Our findings apply only to people who use YouTube fairly regularly and who are likely to encounter information relevant to elections.

That said, it is important to note here that the kind of influence YouTube can exert is especially powerful compared to other common sources of influence that affect people prior to elections. Most of those sources–television advertisements, billboards, and even ballot harvesting–are inherently competitive and often generate relatively small net effects, if any [163].

YouTube, on the other hand, has no effective competitor. Although opposing political campaigns can compete in the process of posting new video content to YouTube, only YouTube controls the process by which those videos are filtered, ordered, and customized. In other words, if employees, executives, or algorithms at YouTube favor one candidate, the opposing candidate has no way of counteracting that favoritism. Similarly, if employees, executives, or algorithms at YouTube choose to suppress the content of one candidate, that candidate has no way to counteract that suppression.

We note that our study and discussion have focused mainly on the potential that a YouTube-like platform has to alter people's thinking and behavior. In our view YouTube presents at least two other major challenges to both researchers and policy makers, and we would be remiss in not pointing them out. First, YouTube has been repeatedly called to task in recent years for what some people consider to be censorship–removing content, restricting access to content, or demoting content on the platform [102, 104, 106–112]. We discussed this issue briefly in our Introduction, referring at one point to a PowerPoint presentation that leaked from Google entitled, "The Good Censor" [18]. Second, YouTube is a surveillance tool; Google openly tracks and subsequently models and monetizes the massive amount of data it collects about the videos people watch and the comments people post on YouTube [164, 165]. The information collected about children has been of special concern to authorities in recent years [166]. The present study needs to be viewed in this larger context. As a powerful and unprecedented tool of surveillance, censorship, and, it seems, manipulation, YouTube should, in our view, be subjected to close scrutiny by both researchers and public policy makers.

Supporting information

S1 Text. Candidate biographies.
(DOCX)

S2 Text. Instructions immediately preceding DoodleTube simulation.
(DOCX)

S3 Text. Vote Manipulation Power (VMP) calculation.
(DOCX)

S1 Table. Experiments 1&2: Demographics.
(DOCX)

S2 Table. Experiments 1&2: VMPs by gender.
(DOCX)

S3 Table. Experiments 1&2: VMPs by race/ethnicity.
(DOCX)

S4 Table. Experiments 1&2: VMPs by educational attainment.
(DOCX)

S5 Table. Experiments 1&2: Mean ratings on the 11-point scale of voting preference for the three groups by gender.
(DOCX)

S6 Table. Experiments 1&2: Mean ratings on the 11-point scale of voting preference for the bias groups (1&2) by gender.
(DOCX)

S7 Table. Experiments 1&2: Mean ratings on the 11-point scale of voting preference for the favored candidate by gender.
(DOCX)

S8 Table. Experiments 1&2: Mean ratings on the 11-point scale of voting preference for the three groups by educational attainment.
(DOCX)

S9 Table. Experiments 1&2: Mean ratings on the 11-point scale of voting preference for Groups 1&2 by educational attainment.
(DOCX)

S10 Table. Experiments 1&2: Mean preference for favored candidate on the 11-point scale of voting preference by educational attainment.
(DOCX)

S11 Table. Experiments 1&2: Mean ratings in the three groups on the 11-point scale of voting preference by race/ethnicity.
(DOCX)

S12 Table. Experiments 1&2: Mean preference shift for Groups 1&2 on the 11-point scale of voting preference by race/ethnicity.
(DOCX)

S13 Table. Experiments 1&2: Mean preference for favored candidate on the 11-point scale of voting preference by race/ethnicity.
(DOCX)

S14 Table. Experiment 1: Pre and Post opinion ratings of favored and non-favored candidates by race/ethnicity.
(DOCX)

S15 Table. Experiment 2: Pre and Post opinion ratings of favored and non-favored candidates by race/ethnicity.
(DOCX)

Acknowledgments

This paper is based on part on a paper presented at the April 2022 meeting of the 102nd annual meeting of the Western Psychological Association. We thank J. Krikorian, H. G. Park, and X. Tong for help with preparing the experiments, and M. Kang, M. Voillot, and V. R. Zankich for help with the data analysis and manuscript preparation. We are especially grateful to L. Kafader and Z. Perez for expert programming assistance.

Author Contributions

Conceptualization: Robert Epstein.

Data curation: Robert Epstein.

Formal analysis: Robert Epstein.

Methodology: Robert Epstein.

Project administration: Robert Epstein.

Supervision: Robert Epstein.

Writing – original draft: Robert Epstein.

Writing – review & editing: Robert Epstein.

References

1. Epstein R, Voillot M. The Video Manipulation Effect (VME): The power that bias in YouTube's up-next algorithm has to shift votes and opinions, and preliminary evidence that such bias exists. Paper presented at: Western Psychological Association; 2022 Apr; Portland, OR. Available from: https://aibrt.org/downloads/EPSTEIN_&_VOILLOT_2022-WPA-YouTube_Manipulation_Effect-VME.pdf
2. Epstein R, Robertson RE. The search engine manipulation effect (SEME) and its possible impact on the outcomes of elections. Proceedings of the National Academy of Sciences of the United States of America. 2015 Aug 4; 112(33):E4512– E4521. Available from: https://www.pnas.org/doi/full/10.1073/pnas.1419828112 PMID: 26243876
3. Epstein R. Why Google poses a serious threat to democracy, and how to end that threat. Congressional Record of the United States. 2019 July 16. Available from: https://www.judiciary.senate.gov/imo/media/doc/Epstein%20Testimony.pdf
4. Epstein R. Manipulating minds: The power of search engines to influence votes and opinions. In: Moore M, Tambini D, editors. Digital dominance: The power of Google, Amazon, Facebook, and Apple. Oxford, UK: Oxford University Press; 2018. pp. 294–319. Available from: https://aibrt.org/downloads/EPSTEIN_2018-Manipulating_minds-The-power_of_search_engines_to_influence_votes_and_opinions-UNCORRECTED_PROOFS.pdf
5. Epstein R, Lee V, Mohr R, Zankich VR. The answer bot effect (ABE): A powerful new form of influence made possible by intelligent personal assistants and search engines. PLOS ONE, 2022 June 1; 17(6): e0268081. https://doi.org/10.1371/journal.pone.0268081 PMID: 35648736
6. Epstein R, Ding M, Mourani C, Olson E, Robertson RE, Tran F. Multiple searches increase the impact of the Search Engine Manipulation Effect (SEME). Paper presented at: 97th annual meeting of the Western Psychological Association; 2017 Apr; Sacramento, CA. Available from: https://aibrt.org/downloads/EPSTEIN_et_al._2017-WPAMultiple_Searches_Increase_the_Impact_of_the_Search_Engine_Manipulation_Effect.pdf
7. Epstein R, Tyagi C, Wang H. What would happen if Twitter sent consequential messages to only a strategically important subset of users? A quantification of the Targeted Messaging Effect (TME). PLOS ONE 18(7): e0284495. https://doi.org/10.1371/journal.pone.0284495 PMID: 37498911
8. McKinnon JD, McMillan D. Google Workers Discussed Tweaking Search Function to Counter Travel Ban: Company says none of proposed changes to search results were ever implemented. The Wall Street Journal. 2018 Sept 20. Available from: https://www.wsj.com/articles/google-workers-discussed-tweaking-search-function-to-counter-travel-ban-1537488472
9. Epstein R. How Google shifts votes: A "go vote" reminder is not always what you think it is. The Epoch Times. 2019 Jan 2. Available from: https://www.theepochtimes.com/another-way-google-manipulates-votes-without-us-knowing-a-go-vote-reminder-is-not-what-you-think-it-is_2754073.html
10. Rosenberg M, Frenkel S. Facebook's role in data misuse sets off storms on two continents. The New York Times. 2018 Mar 18. Available from: https://www.nytimes.com/2018/03/18/us/cambridge-analytica-facebook-privacy-data.html
11. Gottlieb C. The French Competition Authority fines Google €220 million for favoring its own advertising technologies. 7 June 2021. In: Cleary Antitrust Watch. Available from: https://www.clearyantitrustwatch.com/2021/06/the-french-competition-authority-fines-google-e220-million-for-favoring-its-own-advertising-technologies/
12. Gottlieb C. The Commission fines Google €1.49 billion for breaching EU antitrust rules in the Google AdSense investigation. 20 March 2019. In: Cleary Antitrust Watch. Available from: https://www.clearyantitrustwatch.com/2019/03/the-commission-fines-google-e1-49-billion-for-breaching-eu-antitrust-rules-in-the-google-adsense-investigation/
13. Bond RM, Fariss CJ, Jones JJ, Kramer ADI, Marlow C, Settle JE, et al. A 61-million-person experiment in social influence and political mobilization. Nature. 2012 Sept 12. https://doi.org/10.1038/nature11421 PMID: 22972300
14. Kulwin N. The Internet Apologizes. . . Even those who designed our digital world are aghast at what they created. A breakdown of what went wrong—from the architects who built it. Intelligencer. 2018 Apr 16. Available from: http://nymag.com/intelligencer/2018/04/an-apology-for-the-internet-from-the-people-who-built-it.html

15. McNamee R. I invested early in Google and Facebook. Now they terrify me. USA Today. 2017 Aug 8; Available from: https://eu.usatoday.com/story/opinion/2017/08/08/my-google-and-facebook-investments-made-fortune-but-now-they-menace/543755001/
16. Savov V. Google's Selfish Ledger Is an Unsettling Vision of Silicon Valley Social Engineering. The Verge. 2018 May 17. Available from: https://www.theverge.com/2018/5/17/17344250/google-x-selfish-ledger-video-data-privacy
17. Epstein R. Transcript to Google's internal video, "The Selfish Ledger." 2018 May 25. Available from: https://aibrt.org/downloads/GOOGLE-Selfish_Ledger-TRANSCRIPT.pdf
18. Bokhari A. The Good Censory: Google Briefing Accused Trump of Spreading 'Conspiracy Theory'. Breitbart News. 2018 Oct 9. Available from: https://www.breitbart.com/tech/2018/10/09/the-good-censor-google-trump-conspiracy/
19. Vogels EA, Gelles-Watnick R, Massarat N. Teens, social media and technology 2022. Pew Research Center: Internet, Science & Tech. 2022. Available from: https://www.pewresearch.org/internet/2022/08/10/teens-social-media-and-technology-2022/
20. Weill K. How YouTube built a radicalization machine for the far-right. The Daily Beast. 2018 Dec 17. Available from: https://www.thedailybeast.com/how-youtube-pulled-these-men-down-a-vortex-of-far-right-hate
21. Kaushal R, Saha S, Bajaj P, Kumaraguru P. KidsTube: Detection, characterization and analysis of child unsafe content & promoters on YouTube. In: 2016 14th Annual Conference on Privacy, Security and Trust (PST), Auckland, New Zealand, pp. 157–164; 2017 Apr 24. Available from: https://doi.org/10.1109/PST.2016.7906950
22. Papadamou K, Papasavva A, Zannettou S, Blackburn J, Kourtellis N, Leontiadis I, et al. Disturbed YouTube for kids: Characterizing and detecting inappropriate videos targeting young children. The Proceedings of the Fourteenth International AAAI Conference on Web and Social Media. 2020 July 1. Available from: https://doi.org/10.5281/zenodo.363278
23. Auxier B, Anderson M, Perrin A, Turner E. 2. Parental views about YouTube. Pew Research Center: Internet, Science & Tech. 2020 July 28.Available from: https://www.pewresearch.org/internet/2020/07/28/parental-views-about-youtube/
24. Subedar A, Yates W. The disturbing YouTube videos that are tricking children. BBC. 2017 March 27. Available from: http://www.bbc.com/news/blogs-trending-39381889
25. Koerber, B. Gaming the system: How creepy YouTube channels trick kids into watching violent videos. Mashable. 2017 October 22. Available from: http://mashable.com/2017/10/22/youtube-kids-app-violent-videos-seo-keywords/#fRMwn0UO1aqm
26. Mole, B. Suicide instructions spliced into kids' cartoons on YouTube and YouTube Kids: "Sideways for attention. Longways for results," a man says in the middle of a cartoon. Ars Technica. 2019 Feb 25. Available from: https://arstechnica.com/science/2019/02/youtube-kids-cartoons-include-tips-for-committing-suicide-docs-warn/
27. Tan L, Ng SH, Omar A, Karupaiah T. What's on YouTube? A Case Study on Food and Beverage Advertising in Videos Targeted at Children on Social Media. 2018 July 1. Available from: https://doi.org/10.1089/chi.2018.0037 PMID: 29985649
28. Roose K. The making of a YouTube radical. The New York times. 2019 Jun 8; Available from: https://www.nytimes.com/interactive/2019/06/08/technology/youtube-radical.html
29. Fisher M, Taub A. How YouTube radicalized Brazil. The New York times. 2019 Aug 12. Available from: https://www.nytimes.com/2019/08/11/world/americas/youtube-brazil.html
30. Fisher M, Taub A. We wanted to know how online radicalization was changing the world. We started with Brazil. The New York times. 2019 Aug 12; Available from: https://www.nytimes.com/2019/08/11/reader-center/brazil-youtube-radicalization.html
31. Jung EJ, Kim S. Suicide on YouTube: Factors engaging viewers to a selection of suicide-themed videos. PLOS ONE. 2021 June 10. 16(6): e0252796. Available from: https://doi.org/10.1371/journal.pone.0252796 PMID: 34111162
32. Rideout V, Robb MB. The common sense census: Media use by kids age zero to eight, 2020. Common Sense Media. 2020 Nov 17. Available from: https://www.commonsensemedia.org/research/the-common-sense-census-media-use-by-kids-age-zero-to-eight-2020
33. Radesky JS, Schaller A, Yeo SL, Robb MB. Young kids and YouTube: How ads, toys, and games dominate viewing. Common Sense Media. 2020 Nov 17. Available from: https://www.commonsensemedia.org/research/young-kids-and-youtube-how-ads-toys-and-games-dominate-viewing
34. Tufekci Z. Opinion YouTube, the Great Radicalizer. The New York times. 2018 Mar 10; Available from: https://www.nytimes.com/2018/03/10/opinion/sunday/youtube-politics-radical.html

35. Tufekci Z. YouTube's recommendation algorithm has a dark side. Scientific American. 2019 Apr 1; Available from: https://www.scientificamerican.com/article/youtubes-recommendation-algorithm-has-a-dark-side/

36. Lewis R. Alternative Influence: Broadcasting the Reactionary Right on YouTube. Data & Society. 2018 Sept 18; 765:146–57. Available from: https://datasociety.net/library/alternative-influence

37. Jordan G, Shorland A (Producers/Directors). The Weekly Episode 9:'The Rabbit Hole' The New York Times. 2019 Aug 9. Available from: https://www.nytimes.com/2019/08/09/the-weekly/what-is-youtube-pushing-you-to-watch-next.html

38. O'Donovan C, Warzel C, McDonald L, Clifton B, Woolf M. We followed YouTube's recommendation algorithm down the rabbit hole. BuzzFeed News. 2019 Jan 24. Available from: https://www.buzzfeednews.com/article/carolineodonovan/down-youtubes-recommendation-rabbithole

39. Douthat R. Opinion The Faults Beyong Our Algorithms The offline roots of online radicalization. The New York times. 2019 Jun 11. Available from: https://www.nytimes.com/2019/06/11/opinion/youtube-radicalization-algorithms.html

40. Appiah KA. YouTube videos brainwashed my father. Can I reprogram his feed? The New York times. 2021 Apr 20. Available from: https://www.nytimes.com/2021/04/20/magazine/youtube-radicalization.html

41. Nicas J. How YouTube drives people to the internet's darkest corners. Wall Street journal. 2018 Feb 7; Available from: https://www.wsj.com/articles/how-youtube-drives-viewers-to-the-internets-darkest-corners-1518020478

42. Ledwich M, Zaitsev A. Algorithmic extremism: Examining YouTube's rabbit hole of radicalization. First Monday. 24 Dec 2019; Available from: https://firstmonday.org/ojs/index.php/fm/article/view/10419

43. O'Callaghan D, Greene D, Conway M, Carthy J, Cunningham P. Down the (white) rabbit hole: The extreme right and online recommender systems: The extreme right and online recommender systems. Social Science Computer Review. 2015; 33(4):459–78. 2014 Oct 16. Available from: https://doi.org/10.1177/0894439314555329

44. Young HSW, Caine J (Chair/ Member). Report of the Royal Commission of Inquiry into the Attack on Christchurch Mosques on 15 March 2019. The report. 2020 Dec 8. Available from: https://christchurchattack.royalcommission.nz/the-report/

45. Chen AY, Nyhan B, Jason R, Robertson RE, Wilson C. Exposure to Alternative & Extremist Content on YouTube. ADL. 3 May 2022. Available from: https://www.adl.org/resources/report/exposure-alternative-extremist-content-youtube

46. Ribeiro MH, Ottoni R, West R, Almeida VAF, Meira W. Auditing radicalization pathways on YouTube. In: Proceedings of the Conference on Fairness, Accountability, and Transparency; 2020 Jan; Available from: https://doi.org/10.1145/3351095.3372879

47. Alfano M, Fard AE, Carter JA, Clutton P, Klein C. Technologically scaffolded atypical cognition: the case of YouTube's recommender system. Synthese. 2021; 199(1–2):835–58. Available from: https://doi.org/10.1007/s11229-020-02724-x

48. Faddoul M, Chaslot G, Farid H. A longitudinal analysis of YouTube's promotion of conspiracy videos. arXiv. 2020 March 6. Available from: http://arxiv.org/abs/2003.03318

49. Munger K, Phillips J. A Supply and Demand Framework for YouTube Politics. The International Journal of Press/Politics. 27(1). 2019 Oct 1. Available from: https://doi.org/10.1177/1940161220964767

50. Hosseinmardi H, Ghasemian A, Clauset A, Mobius M, Rothschild DM, Watts DJ. Examining the consumption of radical content on YouTube. Proceedings of the National Academy of Sciences of the United States of America. 2021 August 2. Available from: https://doi.org/10.1073/pnas.2101967118 PMID: 34341121

51. Costello M, Hawdon J, Ratliff T, Grantham T. Who views online extremism? Individual attributes leading to exposure. Computers in Human Behavior. 2016 May 13; 63:311–20. Available from: https://doi.org/10.1016/j.chb.2016.05.033

52. Conway M, McInerney L. Jihadi video and auto-radicalisation: Evidence from an exploratory YouTube study. In: Intelligence and Security Informatics. Berlin, Heidelberg: Springer Berlin Heidelberg; 2008. p. 108–18. Available from: https://doi.org/10.1007/978-3-540-89900-6_13

53. Agarwal S, Sureka A. A focused crawler for mining hate and extremism promoting videos on YouTube. In: Proceedings of the 25th ACM conference on Hypertext and social media. New York, NY, USA: ACM; 2014.

54. Ottoni R, Cunha E, Magno G, Bernardina P, Meira WJ, Almeida V. Analyzing right-wing YouTube channels: Hate, Violence and Discrimination. In: Proceedings of the 10th ACM Conference on Web Science. New York, NY: ACM; 2018. Available from: https://arxiv.org/abs/1804.04096

55. Charles C. (Main)streaming hate: Analyzing White Supremacist Content and Framing Devices on YouTube. University of Central Florida; 2020. Available from: https://stars.library.ucf.edu/etd2020/27/

56. Clark S, Zaitsev A. Understanding YouTube Communities via Subscription-based Channel Embeddings. arXiv [cs.LG]. 2020 Oct 19. Available from: http://arxiv.org/abs/2010.09892

57. Hussain MN, Tokdemir S, Agarwal N, Al-Khateeb S. Analyzing disinformation and crowd manipulation tactics on YouTube. In: 2018 IEEE/ACM International Conference on Advances in Social Networks Analysis and Mining (ASONAM). IEEE; 2018. Available from: https://doi.org/10.1109/ASONAM.2018.8508766

58. Statista. Most viewed YouTube videos worldwide 2023. Statista.com. Available from: https://www.statista.com/statistics/249396/top-youtube-videos-views/

59. Wise J. How many videos are uploaded to YouTube A day in 2023? EarthWeb. 2023 May 24. Available from: https://earthweb.com/how-many-videos-are-uploaded-to-youtube-a-day/

60. Schindler P. Expanded safeguards for advertisers. Google. 2017 March 21. Available from: https://blog.google/technology/ads/expanded-safeguards-for-advertisers/

61. YouTube Help. Advertiser-friendly content guidelines. Google.com. 2023. Available from: https://support.google.com/youtube/answer/6162278?hl=en

62. Papadamou K, Zannettou S, Blackburn J, De Cristofaro E, Stringhini G, Sirivianos M. "It is just a flu": Assessing the Effect of Watch History on YouTube's Pseudoscientific Video Recommendations. arXiv [cs.CY]. 2021 Oct 12. Available from: http://arxiv.org/abs/2010.11638

63. Davidson J, Liebald B, Liu J, Nandy P, Van Vleet T, Gargi U, et al. The YouTube video recommendation system. In: Proceedings of the fourth ACM conference on Recommender systems. New York, NY, USA: ACM; 2010.

64. Covington P, Adams J, Sargin E. Deep neural networks for YouTube recommendations. In: Proceedings of the 10th ACM Conference on Recommender Systems. New York, NY, USA: ACM; 2016. Available from: https://doi.oeg/10.1145/2959100.2959190

65. Zuckerberg M. A blueprint for content governance and enforcement. 2018 Nov 15. Facebook. https://perma.cc/ZK5C-ZTSX?type=standard

66. Matamoros-Fernandez A, Gray EJ, Bartolo L, Burgess J, Suzor N. What's 'up next'? Investigating algorithmic recommendations on YouTube across issues and over time. Media and Communication, 9(4), 234–249. 2021 Nov 18. Available from: https://doi.org/10.17645/mac.v9i4.4184

67. Allgaier J. Science and environmental communication on YouTube: Strategically distorted communications in online videos on climate change and climate engineering. Front Commun. 2019; 4. Available from: https://doi.org/10.3389/fcomm.2019.00036

68. Song MY-J, Gruzd A. Examining sentiments and popularity of pro- and anti-vaccination videos on YouTube. In: Proceedings of the 8th International Conference on Social Media & Society—#SMSociety17. NewYork, New York, USA: ACM Press; 2017. Available from: https://doi.org/10.1145/3097286.3097303

69. Bessi A, Zollo F, Del Vicario M, Puliga M, Scala A, et al. Users Polarization on Facebook and Youtube. PLOS ONE 11(8): e0159641. 23 August 2016. Available from: https://doi.org/10.1371/journal.pone.0159641 PMID: 27551783

70. Hussein E, Juneja P, Mitra T. Measuring misinformation in video search platforms: An audit study on YouTube. Proceedings of the ACM Human-Computer Interaction.4(CSCW1):1–27. 2020 May 29. Available from: http://dx.doi.org/10.1145/3392854

71. Roth C, Mazières A, Menezes T (2020). Tubes and bubbles topological confinement of YouTube recommendations. PLOS One, 15(4): e0231703. https://doi.org/10.1371/journal.pone.0231703 PMID: 32315329

72. Airoldi M, Beraldo D, Gandini A. (2016). Follow the algorithm: An exploratory investigation of music on YouTube. Poetics, 57, 1–13. https://doi.org/10.1016/j.poetic.2016.05.001

73. Pariser E. The Filter Bubble. The Penguin Press. 2012 Apr 24. ISBN: 9780143121237.

74. Jamieson KH, Cappella JN. Echo Chambers: Rush Limbaugh and the Conservative Media Establishment. Oxford University Press. https://doi.org/ https://doi.org/10.5860/choice.47-0104

75. Sunstein CR. Echo Chambers: Bush v. Gore, impeachment, and beyond. Princeton, NJ: Princeton University Press. Available from: https://pup.princeton.edu/sunstein/echo.pdf

76. Cho J, Ahmed S, Hilbert M, Liu B, Luu J. Do search algorithms endanger democracy? An experimental investigation of algorithm effects on political polarization. J Broadcast Electron Media. 2020; 64 (2):150–72. Available from: https://doi.org/10.1080/08838151.2020.1757365

77. Hilbert M, Ahmed S, Cho J, Liu B, Luu J. Communicating with algorithms: A transfer entropy analysis of emotions-based escapes from online echo chambers. Commun Methods Meas. 2018; 12(4):260–75. Available from: https://doi.org/10.1080/19312458.2018.1479843

78. Flaxman S, Goel S, Rao JM. Filter bubbles, echo chambers, and online news consumption. Public Opin Q. 2016; 80(S1):298–320. Available from: https://doi.org/10.1093/poq/nfw006

79. Zhao Z, Hong L, Wei L, Chen J, Nath A, Andrews S, et al. Recommending what video to watch next: A multitask ranking system. In: Proceedings of the 13th ACM Conference on Recommender Systems. New York, NY, USA: ACM; 2019

80. Rieder B, Matamoros-Fernández A, Coromina Ò. From ranking algorithms to 'ranking cultures': Investigating the modulation of visibility in YouTube search results. Convergence International Journal of Research into New Media Technologies. 2018; 24(1):50–68. Available from: https://doi.org/10.1177/1354856517736982

81. Kaiser J, Rauchfleisch A. (2020). Birds of a feather get recommended together: Algorithmic homophily in YouTube's channel recommendations in the United States and Germany. *Social Media + Society*, 6 (4), 1–15. https://doi.org/10.1177/2056305120969914

82. Geschke D, Lorenz J, Holtz P. The triple-filter bubble: Using agent-based modelling to test a meta-theoretical framework for the emergence of filter bubbles and echo chambers. Br J Soc Psychol. 2019; 58 (1):129–49. Available from: https://doi.org/10.1111/bjso.12286 PMID: 30311947

83. Bruns A. Echo chamber? What echo chamber? Reviewing the evidence. Snurb.info. 2017. Available from: https://snurb.info/files/2017/Echo%20Chamber.pdf

84. Plasticity Inc. Political disinformation campaign on YouTube–Plasticity. Plasticity.ai. Available from: https://media.plasticity.ai/youtube-disinformation-report/

85. O'Sullivan, Donie. How fake accounts pushing inflammatory content went viral—with the help of YouTube's algorithms. CNN. 2019 Dec 13. Available from: https://edition.cnn.com/2019/12/13/tech/youtube-fake-accounts-viral/index.html

86. Chris A. Top 10 search engines in the world (2023 update). reliablesoft.net. Reliablesoft Academy; 2017 Sept 10. Available from: https://www.reliablesoft.net/top-10-search-engines-in-the-world/

87. Securities and Exchange Commission. Amendment No. 7 to Forms S-1 Registration Statement under The Securities Act of 1933. Securities and Exchange Commission, Washington, D.C. 2004 Aug 13. Available from: https://www.sec.gov/Archives/edgar/data/1288776/000119312504139655/ds1a.htm#toc59330_25b

88. Morris B. The New Tech Avengers: An unlikely triumvirate of Silicon Valley insiders is holding the industry accountable on privacy and addiction. Wall Street Journal. 2018 Jun 29. Available from: https://www.wsj.com/articles/the-new-tech-avengers-1530285064

89. Lewis P. 'Our minds can be hijacked': the tech insiders who fear a smartphone dystopia: Google, Twitter and Facebook workers who helped make technology so addictive are disconnecting themselves from the internet. The Guardian. 2016 October 6. Available from: https://www.theguardian.com/technology/2017/oct/05/smartphone-addiction-silicon-valley-dystopia

90. Moghavvemi S, Sulaiman AB, Jaafar NIB, Kasem N. Facebook and YouTube addiction: The usage pattern of Malaysian students. 2017 July. *2017 International Conference on Research and Innovation in Information Systems (ICRIIS)*, 1–6. http://dx.doi.org/10.1109/ICRIIS.2017.8002516

91. Balakrishnan J, Griffiths MD. Social media addiction: What is the role of content in YouTube? 2017 September 14. *Journal of Behavioral Addictions*, 6(3), 364–377. https://doi.org/10.1556/2006.6.2017.058 PMID: 28914072

92. YouTube. Autoplay. YouTube.com. Available from: https://www.youtube.com/howyoutubeworks/user-settings/autoplay/

93. YouTube Help. Discovery and performance FAQs. Google.com. 2023. Available from: https://support.google.com/youtube/answer/141805?hl%84=%84en

94. Guan Z, Cutrell E. An eye tracking study of the effect of target rank on web search. In: Proceedings of the SIGCHI Conference on Human Factors in Computing Systems. New York, NY, USA: ACM; 2007. Available from: https://doi.org/10.1145/1240624.1240691

95. Pan B, Hembrooke H, Joachims T, Lorigo L, Gay G, Granka L. In Google we trust: Users' decisions on rank, position, and relevance. J Comput Mediat Commun. 2007; 12(3):801–23. Available from: https://doi.org/10.1111/j.1083-6101.2007.00351.x

96. Lewis P. Fiction is outperforming reality: how YouTube's algorithm distorts truth. The guardian. 2018 Feb 2; Available from: https://www.theguardian.com/technology/2018/feb/02/how-youtubes-algorithm-distorts-truth

97. Yang S, Brossard D, Scheufele DA, Xenos MA. The science of YouTube: What factors influence user engagement with online science videos? PLoS One. 2022; 17(5):e0267697. Available from: https://doi.org/10.1371/journal.pone.0267697 PMID: 35613095

98. Meyerson E. YouTube now: Why we focus on watch time. blog.youtube. YouTube Official Blog; 2012. Available from: https://blog.youtube/news-and-events/youtube-now-why-we-focus-on-watch-time/

99. Solsman JE. YouTube's AI is the puppet master over most of what you watch. CNET. 2018 Jan 10. Available from: https://www.cnet.com/tech/services-and-software/youtube-ces-2018-neal-mohan/

100. Warzel C, Smidt R. YouTubers made hundreds of thousands off of bizarre and disturbing child content. BuzzFeed. 2017. Available from: https://www.buzzfeed.com/charliewarzel/youtubers-made-hundreds-of-thousands-off-of-bizarre-and?s=mobile_app&utm_term=.ed3vJl8Kw

101. Fredenburg JN. YouTube as an ally of convenience: The platform's building and breaking with the LGBTQ+ community. Digital Georgetown. Georgetown University; 2020. Available from: http://hdl.handle.net/10822/1059448

102. Google Support. YouTube channel monetization policies. Google.com. Available from: https://support.google.com/youtube/answer/1311392?hl=en&ref_topic=9153642

103. The YouTube Team. YouTube creator blog. YouTube Creator Blog. 2019 Aug 27. Available from: https://youtube-creators.googleblog.com/2019/08/

104. Kumar S. The algorithmic dance: YouTube's Adpocalypse [sic] and the gatekeeping of cultural content on digital platforms. Internet Policy Review. 2019; 8(2). Available from: https://doi.org/10.14763/2019.2.1417

105. Rieder B, Coromina Ò, Matamoros-Fernández A. (2020). Mapping YouTube. *First Monday*. https://doi.org/10.5210/fm.v25i8.10667

106. PragerU. PragerU takes legal action against Google and YouTube for discrimination. Prageru.com. Available from: https://www.prageru.com/press-release-prager-university-prageru-takes-legal-action-against-google-and-youtube-discrimination

107. Neidig, Harper. PragerU sues Google, YouTube for 'censoring' conservative videos. The Hill. 2017 October 24. Available from: http://thehill.com/policy/technology/356966-prageru-sues-google-youtube-for-censoring-conservative-videos

108. Epstein R. The New Censorship: How did Google become the internet's censor and master manipulator, blocking access to millions of websites? Usnews.com. 2016 June 22. Available from: https://www.usnews.com/opinion/articles/2016-06-22/google-is-the-worlds-biggest-censor-and-its-power-must-be-regulated

109. The YouTube Team. The Four Rs of Responsibility, Part 2: Raising authoritative content and reducing borderline content and harmful misinformation. blog.youtube. YouTube Official Blog; 2019 Dec 3. Available from: https://blog.youtube/inside-youtube/the-four-rs-of-responsibility-raise-and-reduce/

110. YouTube Help. Troubleshoot video takedowns. Google.com. 2023. Available from: https://support.google.com/youtube/answer/6395024?hl=en

111. Yourieff, Kaya. (2018, Apr 23). YouTube took down more than 8 million videos in 3 months. CNN. http://money.cnn.com/2018/04/23/technology/youtube-community-guidelines-report/index.html

112. Cruz T. CBS News Uncovers More Big Tech Bias. Ted Cruz. 2019 Dec 3. Available from: https://www.cruz.senate.gov/?p=press_release&id=4795

113. Rankovic Didi. Leaked video shows YouTube CEO talking news manipulation, deciding what's "authoritative" and what's "trashy." Reclaim the Net. 2019 August 17. Available from: https://reclaimthenet.org/youtube-ceo-susan-wojcicki-news-bias

114. Graziosi G. YouTube removes Robert F Kennedy Jr video featuring bizarre claim that polluted water makes children transgender. Independent. 2023 June 19. Available from: https://www.independent.co.uk/news/world/americas/us-politics/youtube-removes-rfk-conspiracy-video-b2360440.html

115. Duffy, C. YouTube removed video of Robert F. Kennedy, Jr. for violating vaccine misinformation policy. CNN. 2023 June 20. Available from: https://www.cnn.com/2023/06/20/tech/youtube-robert-f-kennedy-jr-video-removed/index.html

116. Studio Themes. E-ventures Worldwide LLC vs Google—Google wins the right to de-index. Velocity. 2017. Available from: https://www.velsof.com/blog/ecommerce/e-ventures-worldwide-llc-vs-google-google-wins-the-right-to-de-index

117. United States District Court Middle District of Florida Fort Myers Division. e-ventures Worldwide, LLC Plaintiff, v. Google, Inc., Defendant. Memorandum and Order. 2017. Available from: https://digitalcommons.law.scu.edu/cgi/viewcontent.cgi?referer=&httpsredir=1&article=2410&context=historical

118. Supreme Court of the United States. Reynaldo Gonzalez, et al., Petitioners v. Google LLC, Respondent. Supremecourt.gov. 2023. Available from: https://www.supremecourt.gov/DocketPDF/21/21-1333/254251/20230207191257813_GonzalezRepyMeritsPrinted.pdf

119. McKinnon JD. Google says supreme court ruling could potentially upend the internet. The Wall Street journal (Eastern ed). 2023 Jan 12; Available from: https://www.wsj.com/articles/google-says-supreme-court-ruling-could-potentially-upend-the-internet-11673553968

120. Lutz M, Gadaginmath S, Vairavan N, Mui P. Examining political bias within YouTube search and recommendation algorithms. In: 2021 IEEE Symposium Series on Computational Intelligence (SSCI). IEEE; 2021. Available from: https://doi.org/10.1109/SSCI50451.2021.9660012

121. Shevtsov A, Oikonomidou M, Antonakaki D, Pratikakis P, Ioannidis S. What Tweets and YouTube comments have in common? Sentiment and graph analysis on data related to US elections 2020. 2023 January 31. PLOS ONE 18(1): e0270542. https://doi.org/10.1371/journal.pone.0270542 PMID: 36719868

122. Schwind C, Buder J. Reducing confirmation bias and evaluation bias: When are preference-inconsistent recommendations effective–and when not? Comput Human Behav. 2012; 28(6):2280–90. Available from: https://doi.org/10.1016/j.chb.2012.06.035

123. Agudo U, Matute H. The influence of algorithms on political and dating decisions. PLOS One, 16(4): e0249454. 2021 April 21. Available from: https://doi.org/10.1371/journal.pone.0249454 PMID: 33882073

124. Wittenberg C, Tappin BM, Berinsky AJ, Rand DG. The (minimal) persuasive advantage of political video over text. Proceedings of the National Academy of Sciences of the United States of America. 2021; 118(47):e2114388118. Available from: https://doi.org/10.1073/pnas.2114388118 PMID: 34782473

125. Andreoli V, Worchel S. (1978). Effects of Media, Communicator, and Message Position on Attitude Change. Public Opinion Quarterly, 42(1), 59; Available from: https://doi.org/10.1086/268429

126. Chaiken S, Eagly AH. (1976). Communication modality as a determinant of message persuasiveness and message comprehensibility. Journal of Personality and Social Psychology, 34(4), 605–614. Available from: https://doi.org/10.1037/0022-3514.34.4.605

127. Chaiken S, Eagly AH. (1983). Communication modality as a determinant of persuasion: The role of communicator salience. Journal of Personality and Social Psychology, 45(2), 241–256. Available from https://doi.org/10.1037/0022-3514.45.2.241

128. Horton D, Richard Wohl R. Mass communication and para-social interaction: Observations on intimacy at a distance. Psychiatry. 1956; 19(3):215–29. Available from: https://doi.org/10.1080/00332747.1956.11023049 PMID: 13359569

129. Pfau M, Holbert RL, Zubric SJ, Pasha NH, Lin WK. Role and Influence of Communication Modality in the Process of Resistance to Persuasion. Media Psychology, 2(1), 1–33. 2009 Nov 17.Available from: https://doi.org/10.1207/S1532785XMEP0201_1

130. Weber C, Dunaway J, Johnson T. It's All in the Name: Source Cue Ambiguity and the Persuasive Appeal of Campaign Ads. 34(3), 561–584. 2011 June 2. Available from: https://doi.org/10.1007/s11109-011-9172-y

131. Witus LS, Larson E. A randomized controlled trial of a video intervention shows evidence of increasing COVID-19 vaccination intention. 2022 May 19. PLOS ONE 17(5): e0267580. https://doi.org/10.1371/journal.pone.0267580 PMID: 35587462

132. Arruda W. Why you need to excel at video. Forbes. 2016. Available from: https://www.forbes.com/sites/williamarruda/2016/06/29/why-you-need-to-excel-at-video/

133. Wyzowl. Video marketing statistics 2023. Wyzowl. Available from: https://www.wyzowl.com/video-marketing-statistics/

134. Hong S, Kim N. Will the Internet promote democracy? Search engines, concentration of online news readership, and e-democracy. Journal of Information Technology & Politics, 15(4), 388–399. 2018 October 16. Available from: https://doi.org/10.2139/ssrn.3267049

135. Epstein R. Taming Big Tech: The case for monitoring. Hacker Noon. 2018 May 17. Available from: https://hackernoon.com/taming-big-tech-5fef0df0f00d

136. Epstein R. Ten ways Big Tech can shift millions of votes in the November elections—without anyone knowing. Epoch Times. 2018 September 26. Available from: https://www.theepochtimes.com/10-ways-bigtech-can-shift-millions-of-votes-in-the-november-elections-without-anyoneknowing_2671195.html

137. Epstein R. The unprecedented power of digital platforms to control opinions and votes. In G. Rolnik (Ed.), Digital platforms and concentration: Second annual antitrust and competition conference

(pp. 31–33). Chicago, IL: University of Chicago Booth School of Business; 2018. https://promarket.org/wp-content/uploads/2018/04/Digital-Platforms-and-Concentration.pdf

138. Epstein R, Bock S, Peirson, L, Wang H. Large-scale monitoring of Big Tech political manipulations in the 2020 Presidential election and 2021 Senate runoffs, and why monitoring is essential for democracy. Paper presented at the 24th annual meeting of the American Association of Behavioral and Social Sciences (AABSS). 2021 June 14. Available from: https://aibrt.org/downloads/EPSTEIN_et_al_2021-Large-Scale_Monitoring_of_Big_Tech_Political_Manipulations-FINAL_w_AUDIO.mp4

139. Epstein R, Bock S, Peirson L, Wang H, Voillot M. How we preserved more than 1.5 million online "ephemeral experiences" in the recent US elections, and what this content revealed about online election bias. Paper presented at the 102nd annual meeting of the Western Psychological Association, Portland, OR. 2022 Apr. Available from: https://aibrt.org/downloads/EPSTEIN_et_al_2022-WPAHow_We_Preserved_More_Than_1.5_Million_Online_Ephemeral_Experiences_in_Recent_US_Elections. . .pdf

140. Brandeis LD. Other People's Money, and How the Bankers Use it. 1st ed. Kentucky: Louis D. ho School of Law at the University of Louisville; 1914.

141. Epstein R, Peirson L. How we preserved more than 2.5 million online ephemeral experiences in the 2022 midterm elections, and what this content revealed about online election bias. Paper presented at the 103rd annual meeting of the Western Psychological Association; 2023 Apr; Riverside, CA. Available from: https://aibrt.org/downloads/EPSTEIN_&_Peirson_2023-WPA-How_We_Preserved_More_Than_2.5_Million_Online_Ephemeral_Experiences_in_the_Midterm_Elections.pdf

142. Epstein R. How Google stopped the Red Wave. The Epoch Times. 2022 Nov 15. Available from: https://ept.ms/GoogleRedWave (forwarding link: https://TheMidtermsWereRigged.com)

143. Epstein R. EPSTEIN: Google is shifting votes on a massive scale, but a solution is at hand. Daily Caller. 2022 Nov 6. Available from: https://dailycaller.com/2022/11/06/robert-epstein-2022-midtermelections-google-bing/

144. Epstein R. America's Digital Shield: A new online monitoring system will make Google and other tech companies accountable to the public. Written testimony entered into the Congressional Record. 2023 Dec 13. Available from: https://aibrt.org/downloads/EPSTEIN_2023-Americas_Digital_Shield-Written_Testimony-Senate_Judiciary-13December2023-g.pdf

145. AlgoTransparency. AlgoTransparency. Available from: https://www.algotransparency.org/

146. Lewis P, McCormick E. How an ex-YouTube insider investigated its secret algorithm. The Guardian. 2018 Feb 2. Available from: https://www.theguardian.com/technology/2018/feb/02/youtube-algorithm-election-clinton-trump-guillaume-chaslot

147. Rieder B, Hofmann J. Towards platform observability. Internet Policy Review, 9(4). 18 Dec 2020. Available from: https://doi.org/10.14763/2020.4.1535

148. Agarwal S, Sureka A. Applying social media intelligence for predicting and identifying on-line radicalization and civil unrest oriented threats. arXiv [cs.CY]. 2015. Available from: http://arxiv.org/abs/1511.06858

149. Agarwal N, Gupta R, Singh SK, Saxena V. Metadata based multi-labelling of YouTube videos. In: 2017 7th International Conference on Cloud Computing, Data Science & Engineering—Confluence. IEEE; 2017. Available from: http://doi.org/10.1109/CONFLUENCE.2017.7943219

150. Chaudhary K, Alam M, Al-Rakhami MS, Gumaei A. Machine learning-based mathematical modelling for prediction of social media consumer behavior using Big Data Analytics. Journal of Big Data. 2021; 8(1). https://doi.org/10.1186/s40537-021-00466-2

151. YouTube.

152. Dylko I, Dolgov I, Hoffman W, Eckhart N, Molina M, Aaziz O. The dark side of technology: An experimental investigation of the influence of customizability technology on online political selective exposure. Computer Human Behavior. 2017; 73:181–90. Available from: https://doi.org/10.1016/j.chb.2017.03.031

153. Bai X, Cambazoglu BB, Gullo F, Mantrach A, Silvestri F. Exploiting search history of users for news personalization. Information Sciences, 385–386, 125–137. 2017 April. Available from: https://doi.org/10.1016/j.ins.2016.12.038

154. Bozdag E. Bias in algorithmic filtering and personalization. *Ethics and Information Technology*, 15(3), 209–227. 2013 June 23. Available from: https://doi.org/10.1007/s10676-013-9321-6

155. Loftus EF, Palmer JC. Reconstruction of automobile destruction: An example of the interaction between language and memory 1974. Journal of verbal learning and verbal behavior, 13(5), 585–589.

156. Gravetter FJ, Wallnau LB. Essentials of statistics for the behavioral sciences. 8th ed. Wadsworth Cengage Learning; 2013

157. Lord FM. On the statistical treatment of football numbers. *American Psychologist*, 8(12), 750–751. 1953. Available from: https://doi.org/10.1037/h0063675

158. Townsend JT, Ashby FG. Measurement scales and statistics: The misconception misconceived. Psychological Bulletin, 96(2), 394–401. 1984. Available from: https://doi.org/10.1037/0033-2909.96.2.394

159. Maccoby EE. Parenting and its effects on children: on reading and misreading behavior genetics. Annual Review of Psychology. 2000; 51(1):1–27. Available from: https://doi.org/10.1146/annurev.psych.51.1.1 PMID: 10751963

160. Epstein R., Ding M., Mourani C, Newland A, Olson E, Tran F. Multiple searches increase the impact of similarly biased search results: An example of the Multiple Exposure Effect (MEE). SSRN Electron J. 30 Nov 2023; Available from: https://doi.org/10.2139/ssrn.4636728

161. Yarchi M, Wolfsfeld G, Samuel-Azran T. Not all undecided voters are alike: Evidence from an Israeli election. Government Information Quarterly. 7 October 2021. Available from: https://doi.org/10.1016/j.giq.2021.101598

162. Kaushal N. Top YouTube searches. PageTraffic Blog—All thing Search, Content & Social. PageTraffic; 2023. Available from: https://www.pagetraffic.com/blog/top-youtube-searches/

163. Levitt M. The heyday of television ads is over. Political campaigns ought to act like it. The Washington Post. 2018 November 6. Available from: https://www.washingtonpost.com/outlook/2018/11/06/heyday-television-ads-is-over-political-campaigns-ought-act-like-it/

164. YouTube. How YouTube protects data privacy—How YouTube Works. YouTube.com; 2023. Available from: https://www.youtube.com/howyoutubeworks/our-commitments/protecting-user-data/

165. Charles A. Can YouTube track viewers? Chron.com; 2013. Available from: https://smallbusiness.chron.com/can-youtube-track-viewers-63519.html

166. Brody B, Bergen M. Google to Pay $170 Million for YouTube Child Privacy Breaches. Bloomberg. 2019 Sept 4. Available from: https://www.bloomberg.com/news/articles/2019-09-04/google-to-pay-170-million-for-youtube-child-privacy-breaches
